# Identification of a novel flow-mediated gene expression signature in patients with bicuspid aortic valve

**DOI:** 10.1007/s00109-012-0942-8

**Published:** 2012-08-18

**Authors:** Shohreh Maleki, Hanna M Björck, Lasse Folkersen, Roland Nilsson, Johan Renner, Kenneth Caidahl, Anders Franco-Cereceda, Toste Länne, Per Eriksson

**Affiliations:** 1 Atherosclerosis Research Unit, Center for Molecular Medicine, Department of Medicine, Karolinska Institutet, Stockholm, Sweden; 2 Division of Cardiovascular Medicine, Department of Medical and Health Sciences, Faculty of Health Sciences, Linköping University, Linköping, Sweden; 3 Center for Medical Image Science and Visualization (CMIV), Linköping University, Linköping, Sweden; 4 Computational Medicine, Department of Medicine, Karolinska Institutet, Stockholm, Sweden; 5 Division of Applied Thermodynamics and Fluid Mechanics, Department of Management and Engineering, Linköping University, Linköping, Sweden; 6 Division of Clinical Physiology, Department of Molecular Medicine and Surgery, Karolinska Institutet, Stockholm, Sweden; 7 Cardiothoracic Surgery Unit, Department of Molecular Medicine and Surgery, Karolinska Institutet, Stockholm, Sweden; 8 CMM, L8:03, Karolinska University Hospital, Solna, 171 76 Stockholm, Sweden

**Keywords:** Aortic aneurysm, Transcriptome, Wound healing, Aorta, Thoracic, Congenital heart defects

## Abstract

Individuals with bicuspid aortic valve (BAV) are at significantly higher risk of developing serious aortic complications than individuals with tricuspid aortic valves (TAV). Studies have indicated an altered aortic blood flow in patients with BAV; however, the extent to which altered flow influences the pathological state of BAV aorta is unclear. In the present study, we dissected flow-mediated aortic gene expression in patients undergoing elective open heart surgery. A large collection of public microarray data sets were firstly screened for consistent co-expression with five well-characterized flow-regulated genes (query genes). Genes with co-expression probability of >0.5 were selected and further analysed in expression profiles (127 arrays) from ascending aorta of BAV and TAV patients. Forty-four genes satisfied two filtering criteria: a significant correlation with one or more of the query genes (*R* > 0.40) and differential expression between patients with BAV and TAV. No gene fulfilled the criteria in mammary artery (88 arrays), an artery not in direct contact with the valve. Fifty-five percent of the genes significantly altered between BAV and TAV patients showed differential expression between two identified flow regions in the rat aorta. A large proportion of the identified genes were related to angiogenesis and/or wound healing, with pro-angiogenesis genes downregulated and inhibitory genes upregulated in patients with BAV. Moreover, differential expression of ZFP36, GRP116 and PKD2 was confirmed using immunohistochemistry. Implementing a new strategy, we have demonstrated an angiostatic gene expression signature in patients with BAV, indicating impaired wound healing in these patients, potentially involved in BAV-associated aortopathy.

**Electronic supplementary material:**

The online version of this article (doi:10.1007/s00109-012-0942-8) contains supplementary material, which is available to authorized users.

## Introduction

Bicuspid aortic valve (BAV) is a common congenital disorder with a prevalence of 1–2 % in the population. BAV patients are at higher risk of aneurysm and/or dissection than patients with a tricuspid aortic valve (TAV), even after aortic valve replacement [1].

Two major hypotheses have been put forth to describe the pathogenesis and sensitivity of the proximal aorta to dilation in BAV patients. The ‘genetic theory’ postulates a genetic/ developmental component causing aortic fragility, while the ‘hemodynamic theory’ proposes influence of an abnormal blood flow as a causal factor [2]. Indeed, a collective set of data has demonstrated an abnormal blood flow in the ascending aorta of patients with BAV, with increased flow velocities directed towards the convexity of aortic arch [3, 4]. This particular site coincides with the asymmetrical location of pathological changes in the BAV media [2], suggesting an involvement of flow in the pathology behind BAV aortopathy.

Wall shear stress (WSS) is the frictional force acting on the endothelium as a consequence of blood flow and a key determinant of vascular morphogenesis and vessel physiology in adult life. Exposure of endothelial cells (ECs) to WSS triggers a diverse set of responses, the nature of which encompasses many aspects of cellular metabolism, such as modulation of ion channels, cellular reorganization and changes in cell shape, as well as activation of a set of transcription factors with concomitant activation of their targets [5].

A great deal of research has been carried out measuring gene expression in different types of ECs and vascular smooth muscle cells (VSMCs) exposed to flow in vitro (reviewed in [6]), or by exposing vessels to abnormal blood flow in experimentally manipulated animal models [7, 8]. This has resulted in characterization of a set of shear-responsive genes and identification of Krüppel-like factor 2 (KLF2) as one of the key players in transducing changes in blood flow into EC-specific flow-induced gene expression [6]. KLF2, which has a substantial functional overlap with Krüppel-like factor 4 (KLF4) [9], conveys shear stress signals to the regulation of endothelial homeostasis, establishment of an anti-inflammatory phenotype and induction of vasoprotective genes. Moreover, although the effect of shear stress is primarily sensed by ECs, this effect is further transduced to and sensed by other cell layers in the vessel walls (reviewed in [10]). One example is KLF2 where an EC-specific mutation of *KLF2* changes the vascular tone in embryos [11].

**Fig. 1:**
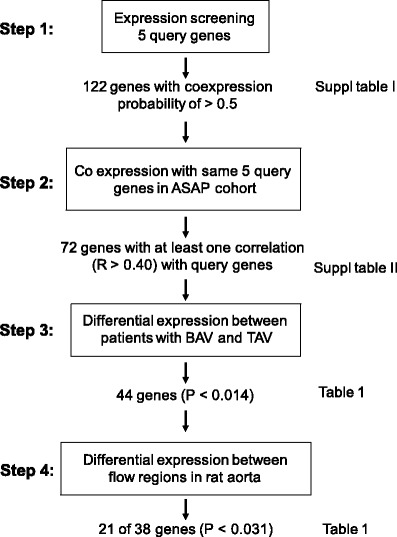
Schematic illustration of data analysis workflow

The purpose of the present study was to investigate whether or not the abnormal blood flow associated with BAV morphology could possibly give rise to a flow-mediated type of gene expression signature specifically associated with BAV phenotype. To study the potential regulatory signals exerted by shear stress on ECs, and in the natural contexts of its interaction with the VSMCs, we used biopsies containing intima–media of the vessel walls of BAV and TAV patients. We firstly used the method ‘expression screening’, which is based on the reasoning that genes showing consistent correlation of mRNA levels across different experimental conditions and in different tissues are likely to be functionally related, in order to identify novel flow-mediated genes. A large collection of public microarray data sets is screened for genes consistently co-expressed with a set of selected well-characterized flow-regulated genes, so-called query genes (step 1, Fig. 1). Genes selected by this procedure were then analysed for co-expression with the query genes in an expression database consisting of array data from intima–media of ascending aorta of BAV and TAV patients (step 2), and the genes that were highly correlated to one or more queries were analysed for association with valve morphology (step 3). To verify that the identified genes are influenced by flow, the mRNA expressions were investigated in regions of rat aortic arch, characterized as being exposed to a disturbed and uniform flow pattern, respectively (step 4). Finally, the protein expression of some genes was evaluated by immunohistochemistry. The study identified novel flow-mediated genes associated with BAV.

## Materials and methods

Detailed experimental protocols are described in the “Supplemental materials”.

### Human material—the ASAP study

The collection of biopsy samples from the Advanced Study of Aortic Pathology (ASAP) database has been described in detail previously [12]. Briefly, biopsies were taken from the anterior side of the ascending aorta, a few centimeters above the valve and from the mammary artery of patients undergoing aortic valve surgery which were having either a BAV or a TAV and dilated or non-dilated ascending aorta. Patients with atherosclerosis (lacking significant stenosis on coronary angiogram) were excluded from the study, and no patients with dissection were included. Demographic data have been presented in detail previously [13]. The study was approved by the regional Human Research Ethics Committee. Oral and written consent was obtained from all patients according to the Declaration of Helsinki.

### Expression screening

The expression screening algorithm has been previously described [14, 15]. Briefly, we used this algorithm to search a repository of 1,512 mouse, rat and human Affymetrix microarray data sets for "informative" data sets where the query genes are co-expressed. Next, a weighted meta-analysis was performed to discover additional genes correlating with the query genes, where the most "informative" data sets are given largest weights. The outcome of this analysis is a Bayesian posterior probability, which reflects the accumulated evidence for co-expression after considering all data sets. We selected genes with a posterior probability greater than 0.5.

### Determination of flow regions in rat

Animal-specific WSS magnitude and vector direction were estimated in the aortic arch of nine male Wistar rats using computational fluid dynamic based on aortic geometry and flow information acquired by magnetic resonance imaging.

### Isolation of RNA

A second set of totally 70 rats was used for analysis of flow-dependent gene expression. In brief, two regions within the same aorta, exposed to a disturbed and uniform flow pattern, respectively, were cut out, and total RNA was isolated using RNeasy Mini Kit.

### Gene arrays

Affymetrix GeneChip Human Exon 1.0 ST arrays, Affymetrix Rat 1.1 ST Titan arrays and protocols were used. Details for transcriptional profiling in the ASAP study have been described previously [12]. Transcriptional profiling of rat aorta was performed on 28 samples (14 pairs of disturbed and uniform flow pattern samples). Microarray results from aortic intima–media were validated by qRT-PCR on 171 samples (127 overlap with microarray cohort) for 11 genes [12].

### Quantitative real-time polymerase chain reaction

Each sample was analysed in triplicates, and standard curve methodology was used for quantification of specific gene targets.

### Immunohistochemistry

Immunostaining was performed on non-dilated aortic section from BAV and TAV patients (*n* = 6 BAV and 6 TAV). Deparaffinised tissue sections (5 μm of thickness) were treated with DIVA solution.

### Statistical analysis

When *P* values are reported (Supplementary Table 2), they are from the association between gene expression and cuspidity, with dilation status, stenosis and regurgitation as covariates. Differential expression of rat RNA microarray data was investigated using a paired Student’s *t* test assuming unequal variance. Correction for multiple testing of gene expression between clinical phenotypes, and between flow regions in rat was done using Benjamini–Hochberg False Discovery Rate correction (FDR) as implemented in the multtest R package. Comparisons between expression of different genes were performed using a Pearson product–moment correlation, with a cutoff of *R* > 0.40.

## Results

### Identifying flow-associated gene expression

Five ‘query genes’ were selected based on thorough evaluation of published data regarding shear-sensitive genes. Special effort was made in order to select well-characterized and well-established shear-sensitive genes, all of which had to be expressed in the vessel wall. KLF2 and KLF4, the main transcription factors specifically induced in response to shear stress in ECs [5]; PKD2, a component of cilia and sensor of shear stress causing autosomal dominant polycystic kidney disease with accompanying cardiovascular phenotypes [16]; thrombomodulin (THBD), a shear stress-induced anti-coagulant associated with aortic remodelling [17]; and tyrosine kinase receptor 1, which is almost exclusively EC-specific and induced in a flow-dependent manner in experimentally manipulated animal vessels [18]. We next used a computational method termed ‘expression screening’ [14, 15] to search a repository of 1,512 mouse, rat and human microarray data sets for where the query genes are co-expressed, and determined which other genes were consistently co-expressed with the query genes in those data sets. Of note, in these data sets, co-expression with query genes is not only in the context of shear stress or mechanosensory activities but also in a wide range of other cellular functions (inevitably, all the queries have several other functions apart from being shear stress responsive). This identified 122 genes with a co-expression probability of >0.5 (Step 1, Fig. 1), presented in Supplementary Table 1a. To check the robustness of the query genes selection, we repeated the procedure with four additional flow-induced genes as queries, namely nitric oxide synthase 3, heme oxygenase 1, SHC-transforming protein 1 and sirtuin 1. This resulted in the selection of 117 genes, all of which were included in the previous list (Supplementary Table 1b).

### Flow-associated gene expression in patients with BAV

We then analysed co-expression of the 122 genes identified by expression screening with the initial 5 query genes in the ASAP microarray data set [12, 13] containing expression profiles from ascending thoracic aorta (127 arrays from intima/media, 81 BAV and 46 TAV; Step 2, Fig. 1). As expected, a large proportion of the genes from step 1 showed co-expression with one or more of the five query genes in the ascending aorta (*n* = 72, Supplementary Table 2a). A Pearson correlation coefficient of 0.40 was used as cutoff for further analyses (FDR of 5 %, *P* < 0.0137 corresponds to *R* = 0.143; Bonferroni correction, *P* < 6.01e−05 corresponds to *R* = 0.404).

In a third step, differential expression between BAV and TAV patients was evaluated using multivariate analysis. To take the effect of other disease phenotypes into consideration, aortic dilatation, regurgitation and stenosis were included as covariates. A cutoff of FDR 5 % (*P* < 0.0140) was used to determine association. Of the 72 genes showing correlation with at least one of the 5 query genes, 44 genes were differentially expressed in the ascending aorta between BAV and TAV patients (Table 1 and Supplementary Table 2a). The putative functions of these genes are listed in Table 1 (including references in Supplementary Table 3). A large proportion of the identified genes were related to angiogenesis and/or wound healing. Further search in PubMed revealed that 89 % (39 out of 44) of the genes have been cited as being induced by flow or overexpression of KLF2 or KLF4 in different cell types (mostly ECs; Table 1). Considering that the query genes function in several other cellular pathways, selection of shear stress-related genes up to 89 % further supported the robustness of filtering procedure. Among the query genes, *KLF2*, *KLF4*, and *PKD2* were differentially expressed between patients with BAV and TAV.

**Table 1:** Genes differentially expressed between flow regions in rat and between BAV and TAV

Gene	RAT		ASAP		Flow	Function(s) and properties
	Disturbed vs uniform flow		BAV vs TAV			
	*P* value	Fold change	*P* value	Fold change		
ZFP36L1	7.80E−07	−1.24^b^	0.0101	−1.64	X*4	Zinc finger protein, modulation and destabilization VEGF mRNA, wound healing
ZFP36	1.08Ev−05	−1.37^b^	2.22E−05	−1.25	X*4	Modulation and destabilization VEGF mRNA, wound healing
FLI1	6.60E−05	1.21	3.66E−06	−1.39	X	Angiogenesis, maturation and stabilization of vessels, wound healing
EGR1	7.98E−05	−1.42^b^	0.000777	−1.31	X	Regulates angiopoietin-1 induced EC migration and proliferation, wound healing
IER2	0.00012	−1.19^b^	0.000429	−1.17	X*4	Mediates EGF-dependent left–right asymmetry patterning in zebrafish
SLC2A3	0.00019	1.36	6.51E−08	−1.44	X*4	Involved in tumour angiogenesis
FOSB	0.00031	−1.51^b^	1.23E−05	−1.42	X	Induced by mechanical stress and cardiac ischemia and VEGF, wound healing
TIMP1	0.00045	1.19	0.00329	−1.17	X	Anti-angiogenesis, wound healing
GRK5	0.00053	1.19^b^	8.30E−05	1.17	X	Regulation of vasoconstriction in VSMC, histone deacetylase (HDAC) kinase in cardiomyocytes
CCL2	0.00093	−1.47^b^	1.32E−06	−1.80	X	Involved in neovascularization and angiogenesis, wound healing
NID1	0.00098	−1.14^b^	0.00197	−1.40	X	Ingredient of vascular basement membranes, wound healing
ITGA5	0.00115	−1.19^b^	9.00E−04	−1.74	X	Fibronectin receptor
SOCS3	0.00119	−1.26^b^	2.84E−07	−1.35	X	Negative regulator of cytokines, wound healing
FOS	0.00139	−1.34^b^	6.31E−05	−1.65	X	Regulation of VEGF, angiogenesis, wound healing
KLF2 ^a^	0.00207	−1.29^b^	0.0105	−1.10	X	Regulates VEGFA, angiogenesis
BTG2	0.00314	−1.15^b^	1.35E−05	−1.30	X	Anti-proliferative transcription factor involved in modulation of VEGF-regulated wound repair
SGK1	0.01066	1.10	0.000461	−1.33	X	Involved in vascular remodelling during angiogenesis, wound healing
PLEKHO2	0.01227	1.10	6.75E−08	−1.25	?	No functional studies performed according to PubMed
PTGER4	0.01418	−1.29^b^	1.92E−06	−1.33	X	Mediates prostaglandin E2 stimulated VEGF expression, angiogenesis, wound healing
COL6A3	0.01504	1.10	9.00E−05	−1.24	X	ECM component, regulated by TGF-β
FERMT2	0.02228	1.10^b^	0.000179	1.17	?	Wound healing, regulated by TGF-β1, angiogenesis
IL1R1	0.03103		0.0104	−1.21	X	Wound healing
PKD2 ^a^	0.04592		7.03E−07	1.30	X	Ca^2+^-permeable channel of cilia, involved in embryonic left right (LR) symmetry, wound healing
GEM	0.13107		0.000193	1.20	X	Regulation of voltage-gated Ca^2+^ channel
DAB2	0.13354		0.000298	−1.30	X*2	Embryonic angiogenesis via VEGF induction, TGF-β-stimulated fibronectin synthesis in wound healing
GPR116	0.18922		1.99E−08	−1.86	X*2	Component of microvasculature
DUSP5	0.27924		8.17E−05	−1.16	X	Strongly induced by VEGF in EC, EC specific in vessels development
CEBPB	0.40525		0.000551	−1.12	?	Interacts directly with subunits of NF-kB to augment gene expression by FOS transcription factors
KLF4^a^	0.44656		8.29E−05	−1.25	X	Modulates phenotype of VSMCs, angiogenesis, wound healing
SH2B3	0.48533		8.03E−07	−1.18	?	Stabilization of thrombi within vessels, regulates EPC kinetics in vascular regeneration
JUNB	0.49141		2.60E−05	−1.40	X	Regulates VEGF expression in an NF-kB-dependent manner, angiogenesis, wound healing
CDH5	0.53465		2.52E−05	−1.23	X	Ingredient of shear stress sensor complex, angiogenesis, EC specific in vessel development
CD93	0.68071		0.000794	−1.22	X*2	Vascular remodelling and angiogenesis
CDKN1A	0.71809		0.002	−1.16	X	Involved in regulation of EC senescence and permeability, wound healing
PTPRE	0.79921		4.96E−07	−1.22	X	Negative regulation of EC proliferation, possible role in angiogenesis
CALD1	0.93732		0.00108	1.17	X*2	Anti-angiogenesis, cytoskeletal protein, implicated in the migration of EPC
ERG	0.96645		0.00718	1.14	X*2	Angiogenesis, wound healing through regulation of ECM
ELTD1	0.99016		3.03E−05	−1.52	X*4	Specifically expressed in the microvasculature
ENG	NA	NA	0.000117	−1.42	X	Vascular TGF-β co-receptor, angiogenesis, wound healing
PECAM1	NA	NA	0.000899	−1.48	X	Ingredient of shear stress sensor complex, angiogenesis, wound healing
CEBPD	NA	NA	0.000296	−1.20	X*4	Interacts with subunits of NF-kB to augment gene expression by FOS transcription factors
THBS1	NA	NA	0.00201	−1.37	X	Anti-angiogenesis, wound healing
KIAA0247	NA	NA	5.25E−05	−1.17	?	Regulator of cell cycle
IL4R	NA	NA	1.23E−06	−1.34	X	IL-4 receptor, IL-4 has been indicated in both promoting and blocking angiogenesis

*P* values for differential expression between disturbed and uniform flow regions in rat aorta and for genes differentially expressed between BAV and TAV are presented

*X* induced by shear stress according to the literature, X*2 by KLF2 or X*4 by KLF4 overexpression (extracted from literature search), *?* not cited in the literature, *NA* not analysed

^a^Query genes

^b^Fold changes for genes moving in the same direction in BAV and the areas of rat aorta exposed to disturbed flow

Gene expression profiles from mammary artery from the same patient group (88 arrays) were used as controls as this vessel is not under the direct influence of flow-mediated effects by the valve. Similar to the ascending aorta, the majority of genes from step 1 correlated with one or more of the five query genes (76 of 122, data not shown), but no gene that correlated with query genes showed differential expression between patients with BAV and TAV at FDR 5 % (Supplementary Table 2b).

**Fig. 2:**
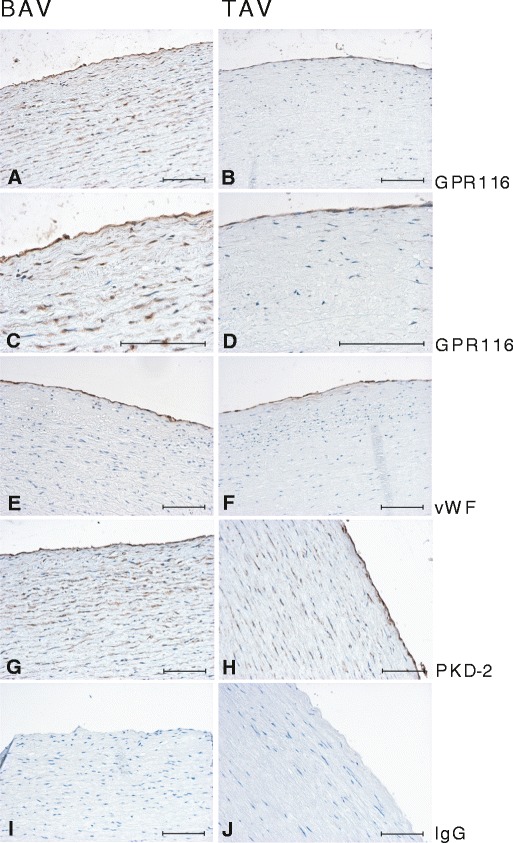
Immunostaining of GPR116 and PKD2 in non-dilated aorta of BAV and TAV patients. **a**, **c** GPR116 staining in BAV; **b**, **d** GPR116 staining in TAV; **e**, **f** vWF staining in BAV and TAV, respectively; **g** PKD2 staining in BAV; **h** PKD2 staining in TAV; **i**, **j** negative control. *Scale bar* = 100 μm

Protein expression of GPR116, the most significantly altered gene at mRNA level between BAV and TAV patients (*P* = 1.99e−08), was analysed by immunohistochemistry in non-dilated ascending aorta of BAV and TAV patients. As seen in Fig. 2, a strong GPR116 expression was detected in ECs of both BAV and TAV patients. In BAV patients, there was also a strong staining in medial VSMCs, as opposed to medial VSMCs from patients with TAV, in which the staining was much weaker. A similar expression pattern was observed for the query gene PKD2 (Fig. 2).

### Expression of flow-associated genes in regions with different flow patterns in rat aorta

Genes identified as differentially expressed between BAV and TAV patients (44 genes after filtering steps 1–3, Fig. 1) were analysed in two regions exposed to different flow patterns in the rat aortic arch; the distal part of the inner curvature of the arch, subjected to a disturbed flow pattern (i.e. a non-uniform WSS vector direction near the wall), and a region along the outer curvature of the arch, directly after the left subclavian artery, exposed to uniform flow fields (i.e. a uniform WSS vector direction near the wall; Step 4, Fig. 1). The regions have been identified in our lab previously (Björck et al. manuscript), and RNA had been extracted and subjected to global gene expression analysis. The array platform included 38 of the 44 identified genes. The mRNA expression of 21 genes (55 %) differed (FDR 5 %, *P* < 0.031) between uniform and disturbed flow pattern regions (Table 1). Of these genes, 15 (71 %) were either up- or downregulated in the same direction in BAV and disturbed flow regions (Table 1).

**Fig. 3:**
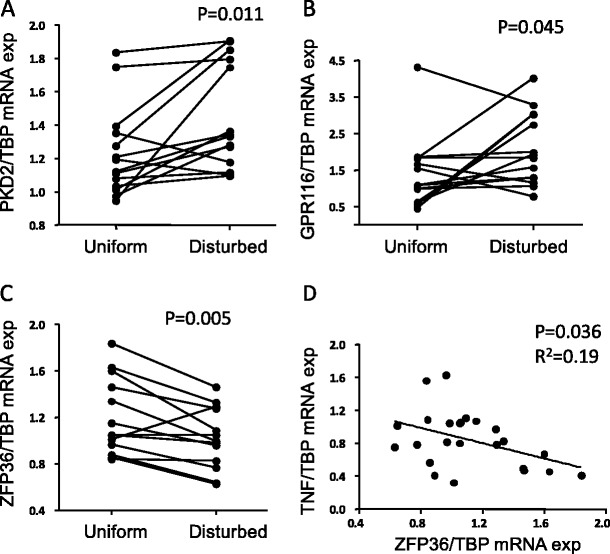
Expression of query and non-query genes in uniform and disturbed flow pattern regions of rat aorta. Shown are *PKD2* (**a**), *GPR116* (**b**) and *ZFP36* (**c**) mRNA expression. **d** Correlation between *ZFP36* and *TNF* mRNA expression (*n* = 26). Gene expression was analysed by quantitative real-time PCR and normalized to TBP mRNA expression

As shown in Fig. 3, *PKD2* had higher mRNA expression in regions exposed to a disturbed flow pattern when analysed with real-time PCR (real-time-PCR *P* = 0.011, gene arrays *P* = 0.0459). Similarly, protein expression of PKD2 was increased in the vessel walls of rat aorta exposed to a disturbed flow pattern compared with areas of uniform flow (Supplementary Fig. 1). Furthermore, we also analysed the expression of TNF and VCAM1, two known disturbed shear stress-induced genes, as controls. Both *TNF* and *VCAM1* were higher in the disturbed flow pattern regions than in the uniform flow pattern regions (*P* = 0.0001 and *P* = 0.002, respectively, Björck et al. manuscript).

The mRNA expression of the *GPR116* showed a trend towards increased mRNA expression in disturbed flow pattern regions (*P* = 0.19). However, there was a significant increase in mRNA expression in regions with a disturbed flow pattern when analysed with real-time PCR (Fig. 3, *P* = 0.045).

### Downregulation of ZFP36 and ZFP36L1 expression in regions of disturbed flow in rat and in aorta of BAV patients

Among the 44 genes significantly altered in the ascending aorta between BAV and TAV patients, the genes showing the largest difference in expression between the two flow pattern regions in rat were *ZFP36* and *ZFP36L1*. ZFP36 and ZFP36L1 are two closely related RNA-binding zinc finger proteins binding to the 3′-untranslated regions of several mRNAs and promote their degradation [19–21]. Both genes showed significantly lower expression in regions with a disturbed flow pattern and a significantly lower expression in BAV compared with TAV patients. The differential expression of *ZFP36* in rat aortic arch was further confirmed using real-time PCR (Fig. 3). ZFP36 has previously been shown to inhibit TNF production by destabilization of its mRNA [19]. As expected, we observed a significant inverse correlation between *TNF* and *ZFP36* expression in rat (*P* = 0.036, *R* ^2^ = 0.19; Fig. 3). In addition, *ZFP36* expression was inversely correlated to *VCAM1* expression (*P* = 0.030; data not shown).

**Fig 4:**
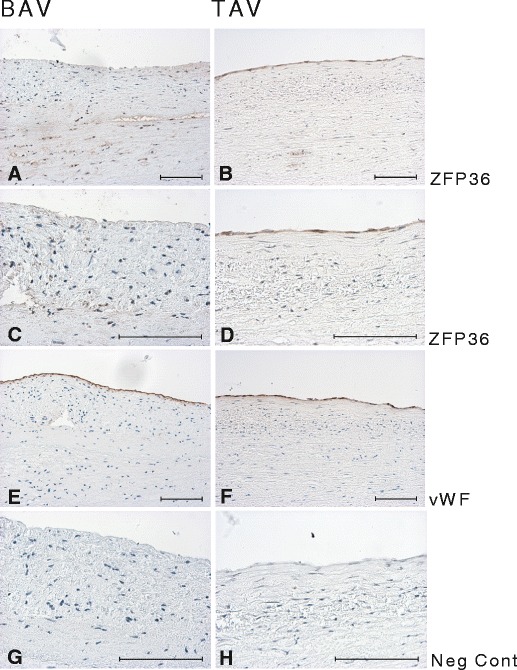
Immunostaining of ZFP36 in non-dilated aorta of BAV (**a**, **c**) and TAV (**b**, **d**) patients. **e**, **f** vWF staining in BAV and TAV, respectively; **g**, **h** negative control. *Scale bar* = 100 μm

Protein expression of ZFP36 was analysed in ascending aorta of BAV and TAV patients. As shown in Fig. 4, ECs in TAV patients expressed ZFP36, whereas endothelial expression of ZFP36 was absent in BAV patients. In agreement with real-time PCR and array expression data, ZFP36 staining was clearly decreased both in ECs and VSMCs in areas exposed to a disturbed flow pattern in rat (Supplementary Fig. 2).

## Discussion

In this report, we analysed the ascending aortic gene expression profile in patients with BAV and TAV from a flow perspective. Using a combination of methods, we described a potential flow-mediated gene expression associated with BAV and disclosed that a large proportion of the identified genes were induced by shear stress and related to angiogenesis and/or wound healing. Importantly, in the same group of patients, no genes were differentially expressed in the mammary artery between BAV and TAV patients when the same selection procedure was applied, suggesting that the identified gene expression pattern was specific for the ascending aorta close to the aortic valve itself. It should be emphasized that the differential expression of these genes is most likely due to the aortic cusp morphology as atherosclerosis patients were excluded and other disease phenotypes, i.e. dilatation, regurgitation and stenosis, were included as covariates in the multivariate analysis. Using a wider set of query genes further confirmed a robust co-expression with queries. Moreover, 55 % of the identified genes were differentially expressed between uniform and disturbed flow in rat aorta.

Shear stress is sensed by ECs via a number of membrane-associated mechanosensors, such as ion channels and growth factor receptors, G protein-coupled receptors, adhesion proteins, the cytoskeleton and primary cilia [6]. Genes selected by our procedure belong to these groups (Table 1). Most importantly, a differential expression of KLF2 and KLF4, the two major flow-induced transcription factors, and PECAM1 and CDH5, components of the mechanosensory complex mediating EC responses to fluid shear stress [22], was observed between BAV and TAV patients.

The gene function literature search, summarized in Table 1, revealed that a large proportion of the genes differentially expressed between BAV and TAV were either directly or indirectly connected to angiogenesis. However, most pro-angiogenesis genes (with the exception of *ERG* and *FERMT2*) were downregulated, while genes with inhibitory effects on angiogenesis (with the exception of *THBS1*) were upregulated in BAV patients, indicating an angiostatic gene expression. As expected, many genes involved in angiogenesis also regulated wound healing processes in different types of tissues. One likely explanation for the downregulation of angiogenesis/wound healing genes in BAV patients may be a general reduction of progenitor cells, as has been reported in BAV patients with non-functional valves [23] and in patients with aortic valve stenosis [24].

Endothelial progenitor cells (EPCs) originate either from the vessel wall adjacent to the damaged vasculature or from the bone marrow, and have been implicated in angiogenesis as well as maintenance of EC integrity, adult neovascularization and wound healing. The association of progenitor cells with cardiovascular disease has been the subject of intensive investigations (for review see [25]), and enhanced proliferation of EPC as a result of exposure to uniform shear stress [26, 27] and overexpression of KLF2 [28] have been reported. It can be hypothesized that exposure of the BAV aorta to abnormal flow results in a decreased local angiogenesis due to reduced number of EPCs, thereby contributing to a diminished repair capacity and fragility of aorta. A similar mechanism has been proposed for difficulties in wound healing in diabetic patients [29]. This hypothesis fits well with our recently published observation demonstrating a decreased expression of EDA splice variant of fibronectin, which is important for tissue repair, in VSMCs isolated from the BAV aorta compared with VSMCs from the TAV aorta [30].

We showed for the first time that ZFP36 and ZFP36L1 were differentially expressed in the ascending aorta between BAV and TAV patients, and that these genes were flow-responsive in the rat aortic arch. ZFP36 was downregulated in BAV aorta and in aortic regions exposed to disturbed flow in rat, both at mRNA and protein level. In BAV patients, the reduction was most prominent in the endothelial layer. ZFP36 and ZFP36L1 regulate the stability of numerous mRNAs, including *TNF*, *VEGF* and *NFKB1* [19–21], and possess anti-inflammatory properties. The downregulation of ZFP36 in BAV may thus seem unexpected as we recently reported an association of TAV morphology with an overexpression of immune response-related genes [12]. The function of this protein in aetiology of BAV is however not known, but may be more associated with the regulation of wound healing-related angiogenesis via regulation of VEGF rather than inflammation (see below).

Abnormal expression of PKD2 in patients with polycystic kidney disease has been associated with a range of vascular abnormalities including BAV morphology [31], intracranial aneurysms, dilatation of the aortic root and dissection of the thoracic aorta [16]. In VSMCs, PKD2 inhibits the activity of stretch-activated ion channels (SACs), and elevated expression of PKD2 is associated with impaired arterial myogenic tone via alteration of PKD1/PKD2 ratio [32]. We observed a marked increase in PKD2 staining of VSMCs in BAV aorta compared with TAV, which may well contribute to impaired VSMC function in BAV aorta. However, EC exposure to disturbed flow may also cause a difference in the expression of PKD2 in the VSMCs. In mice, primary cilia have been shown to be more abundant in regions exposed to low or oscillatory shear stress [33], and ciliated ECs are restricted to areas exposed to disturbed flow in the chick embryonic heart, in which the expression of KLF2 is downregulated [8, 34]. Hence, increased number of endothelial cilia in BAV patients, as a consequence of disturbed flow, may be connected to upregulation of PKD2 in VSMCs. Whether or not disturbed shear stress can differentially regulate SACs in VSMCs directly or indirectly through EC signalling is unknown. Indeed, our results showed that *PKD2* and *KLF2* expression were inversely correlated. That was also true for the expression of majority of the genes correlated to each one (Supplementary Table 1).

GPR116 and ELTD1 have previously been shown to be EC-specific markers and broadly expressed in the vascular endothelium [35]. Our data regarding the expression pattern of GPR116 in association with BAV and perturbed flow are not conclusive. The array results indicated that GPR116 expression was downregulated in the BAV aorta, while immunostaining showed strong GPR116 expression in ECs of both BAV and TAV patients. However, there was a strong GPR116 staining in VSMCs of BAV patients. The discrepancy between mRNA and protein expression is a common phenomenon and may reflect changes in the regulation of translation. Whether or not the absolute amount of GPR116 protein is increased in BAV or only in medial layer is hard to establish by immunostaining, but the pattern of protein distribution clearly differed between BAV and TAV.

Although our filtering successfully identified novel flow-mediated genes associated with valve morphology, there are some disadvantages of the selection procedure. The analysed mRNA contains a disproportionate mixture of EC and VSMC messages, and co-expression analysis with query genes in the ASAP data set will not specify cell origin. However, it is clear from our result that in spite of disproportionate amount of EC and VSMC mRNA, our selection procedure was capable of identifying several EC-specific genes (i.e. *PECAM1*, *CDH5* and several others, Table 1), as well as detecting differential expression of ZFP36 protein in ECs of the two patient groups, whereas in the case of PKD2, the differential expression seems to be more due to the VSMC. Due to the importance of media degeneration for aneurysm development, we believe that this procedure is more likely to capture real alterations in the vessel wall than isolated EC or VSMC grown in culture media. This was elegantly addressed in a study by Li et al. [17] in mice, in which they showed that THBD was limited to the endothelial layer in the normal vessel wall. However, when the vessel was exposed to longer periods of high WSS, VSMCs were activated and the vessel wall remodelled. The medial change was accompanied by appearance of THBD in the medial layer, and as discussed by the authors, this type of vessel changes would not be captured by cell cultures.

In summary, the present study investigated flow-associated gene expression in the ascending aorta of BAV and TAV patients, using for the first time ‘expression screening’ in human material. Taking advantage of a multi-step analysis strategy, several novel genes associated with valve morphology, particularly related to angiogenesis and wound healing, were identified. Importantly, the majority of these genes were subsequently validated as being flow-responsible in the rat aortic arch. Moreover, our results extended our previous findings on fibronectin splicing in VSMCs of BAV patients, and its implication for VSMC repair, to vascular endothelial repair deficiency, most probably laying the foundation for endothelial dysfunction in BAV patients. However, although this work identified several genes that appear to be modulated by BAV-related changes in flow, their involvement in BAV-associated aortopathy remains to be shown.

## Electronic supplementary material

Below is the link to the electronic supplementary material.

ESM 1 (PDF 743 KB)
