# Supplementary material for: Identification of a novel flow-mediated gene expression signature in patients with bicuspid aortic valve
Source: J Mol Med (Berl). 2012 Aug 18;91(1):129–39. doi: 10.1007/s00109-012-0942-8 (PMC3536974; doi:10.1007/s00109-012-0942-8)
Supplement: Supplementary file 1 — (PDF 743 KB) [file 109_2012_942_MOESM1_ESM.pdf]

Supplementary materials

Identification of a novel flow-mediated gene expression signature in patients with bicuspid aortic valve

Shohreh Maleki[1]*, Hanna M Björck[2,3]*, Lasse Folkersen[1], Roland Nilsson[4], Johan Renner[3,5], Kenneth Caidahl[6], Anders Franco-Cereceda[7], Toste Länne[2,3], Per Eriksson[1]

* Equal contribution

[1]Atherosclerosis Research Unit, Center for Molecular Medicine, Department of Medicine, Karolinska Institute, Stockholm, Sweden, [2]Division of Cardiovascular Medicine, Department of Medical and Health Sciences, Faculty of Health Sciences, Linköping University, Sweden, [3]Center for Medical Image Science and Visualization (CMIV), Linköping University, Sweden, [4]Computational Medicine, Karolinska Institute, [5]Division of Applied Thermodynamics and Fluid Mechanics, Department of Management and Engineering, Linköping University, Sweden, [6]Clinical Physiology, [7]Cardiothoracic Surgery Unit, Department of Molecular Medicine and Surgery, Karolinska Institute, Stockholm, Sweden.

MATERIALS AND METHODs

Human Material – the ASAP study

The collection of biopsy samples from the Advanced Study of Aortic Pathology (ASAP) database has been described in detail previously (1). Briefly, biopsy samples were taken from ascending aortas of patients undergoing aortic valve surgery which were classified as having either BAV or TAV and dilated or non-dilated aorta. Demographic data have been presented in detail previously (2).

Animals

Male Wistar rats (Taconic, Lille Skensved, Denmark) weighing 400-450 g were used. The experimental protocol was approved by the Regional Ethics Committee for Animal Experiments, and followed institutional guidelines for care and treatment of experimental animals. Animals were maintained at the Animal Facility, Faculty of Health Sciences, Linköping University under constant environmental temperature (21°C), and a 12h:12h light/dark cycle, and had free access to standard rodent chow and water.

Expression screening

The expression screening algorithm has been previously described (3,4). Briefly, we used this algorithm to search a repository of 1,512 mouse and human Affymetrix microarray data sets for "informative" data sets where the query genes are co-expressed. Next, a weighted meta-analysis was performed to discover additional genes correlating with the query genes, where the most "informative" data sets are given largest weights. The outcome of the analysis is a posterior probability of true co-expression. We selected genes with a posterior probability greater than 0.5.

Determination of flow regions in rat

A total of nine rats were used for determination of flow regions. A detailed description of the examination procedure and methods employed can be found elsewhere (Björck et al. submitted manuscript). In brief, geometry and flow information from the rat aorta were obtained using a 1.5 T whole body magnetic resonance imaging (MRI) scanner and an eight channel wrist coil (Philips Achieva, Philips Medical Systems, Best, the Netherlands). Segmentation of the MRI image data material into a 3D geometry of the aortic arch was performed using a semi-automatic level-set approach, followed by subject specific estimation of time resolved wall shear stress (WSS) magnitude and WSS vector direction by the use of computational fluid dynamics (CFD). The flow was assumed to be laminar and the aortic wall was considered to be rigid with a no slip boundary condition.

Isolation of RNA

A second set of totally 70 rats was used for analysis of gene expression. A detailed description of the experimental procedure is provided elsewhere (Björck et al. manuscript). In brief, two regions within the same aorta, exposed to a disturbed and uniform flow pattern, respectively were cut out and directly put in pre-chilled Lysing Matrix D tubes (MP Biomedicals, Illkirch, France) containing Trizol (Invitrogen, Paisley, Scotland, UK). Tissue from the specific regions from five animals was pooled in order to yield significant amounts of RNA. Samples were homogenized with FastPrep. Total RNA was isolated using RNeasy Mini kit (Qiagen, Maryland, USA) as a cleanup, including DNase treatment for elimination of potential DNA contamination. RNA integrity was analyzed by the use of an Agilent 2100 Bioanalyzer (Agilent Technologies Inc., Paolo Alto, CA, USA) and quantified using NanoDrop (NanoDrop products, Wilmington, DE, USA).

Gene arrays

The RNA samples were hybridized and scanned at the Karolinska Institute microarray core facility. Affymetrix GeneChip Human Exon 1.0 ST arrays, Affymetrix Rat 1.1 ST Titan arrays and protocols were used (Affymetrix, Santa Clara, CA). In both studies the raw cel files were

preprocessed and log2 transformed using Robust Multichip Average normalization as implemented in the Affymetrix Power Tools 1.10.2 package apt-probeset-summarize (5). This normalization includes a step in which the distribution of gene expression levels on individual arrays are standardized, normalizing the expression levels to the overall mRNA levels.

Details for transcriptional profiling in the ASAP study has been described previously (1). Gene annotation for this study was downloaded from the Affymetrix web page as version HuEx-1_0-st-v2.na29.hg18. All investigations were done on the core sets of meta probes provided by Affymetrix. The data from mammary artery intima-media and from ascending thoracic aorta intima-media are used.

Transcriptional profiling of rat aorta was performed on 28 samples (14 pairs of disturbed and uniform flow pattern samples) (Björck HM, et al. manuscript). Gene annotation was downloaded from the Affymetrix web page as version RaGene-1_1-st-v1.na31.rn4.

Quantitative real-time polymerase chain reaction
A total of 200 ng RNA from each sample was reversed transcribed with random primers and Superscript II (Invitrogen, Carlsbad, CA, USA). Amplification of cDNA was performed in 20µl reactions using 1xTaqMan Universal PCR Mastermix (Applied Biosystems, Foster City, CA, USA) on an ABI 7700 Sequence Detector. Each sample was analyzed in triplicates and standard curve method was used for quantification of specific gene targets (for reference see e.g. Cikos, S. et al. *BMC Mol Biol*. 2007;8:113). The following Assay on Demand kits (Applied Biosystems, Foster City, CA, USA) were used: *GPR116*, Rn00594813; *PKD2*, Rn01449962; *TNF-α*, Rn00562055; *VCAM1*, Rn00563627; *ZFP36*, Rn01763978. TATA-binding protein (*TBP*, Rn01455646) served as an RNA loading control. Reactions without template were included as negative controls. The thermal protocol were as follows: 50°C for 2 min, 95°C for 10 min, followed by 45 repeats of 95°C for 15 sec, 60°C for 1 min.

Immunohistochemistry
Immunostaining for PKD-2, ZFP36, GPR116 and vWF was performed on deparaffinised tissue sections treated with DIVA solution (Biocare Medical, Concord, CA) using rabbit-anti-human/mouse/rat PKD-2 polyclonal antibody (ab78622, Abcam, Cambridge, UK), rabbit-anti-human/mouse/rat ZFP36 polyclonal antibody (sc-14030, Santa Cruz, CA), goat-anti-human/mouse/rat GPR116 polyclonal antibody (sc-68978), and rabbit-anti-human vWF polyclonal antibody (A0082, DakoCytomation, Glostrup, Denmark), respectively. Endogenous peroxidase activity was quenched with 3% hydrogen peroxide for 5 min, and nonspecific binding sites were blocked with 20% goat or horse serum. Biotinylated anti-rabbit and anti-goat IgG (Vector, Peterborough, UK) was used as secondary antibody. Normal rabbit IgG (sc-2027, Santa Cruz, CA), normal goat IgG (sc-2028, Santa Cruz, CA) and PBS was included as controls. Avidin-biotin peroxidase complex (Vectastain ABC kit, Vector Laboratories, Burlingame, CA) was added for 30 min in room temperature, followed by visualization using 3,3'-diaminobenzidine tetrahydrochloride (Dako, Glostrup, Denmark). All sections were counterstained with Mayer's hematoxylin (Histolab Products, Göteborg, Sweden).

Statistical analysis
Multivariate analysis models refer to the use of the linear model lm function as implemented in R 2.13.0. When P-values are reported (supplementary table II), they are from the association between gene expression and cuspidity, with dilation status, stenosis and regurgitation included in the model. Differential expression of rat RNA microarray data was investigated using a paired Student's T-test assuming unequal variance. Correction for multiple testing of gene expression between clinical phenotypes, and between flow regions in rat was done using Benjamini-Hochsberg False Discovery Rate correction (FDR) as implemented in the *multtest* R-package. Comparisons between the expression of different genes were performed using a Pearson product-moment correlation, with a cutoff of R >0.40.

Supplementary Fig 1

Immunostaining of PDK2 in disturbed (A) and uniform (B) flow pattern regions of rat aorta. C and D, vWF staining in disturbed and uniform plow pattern regions, respectively; E and F, negative control.

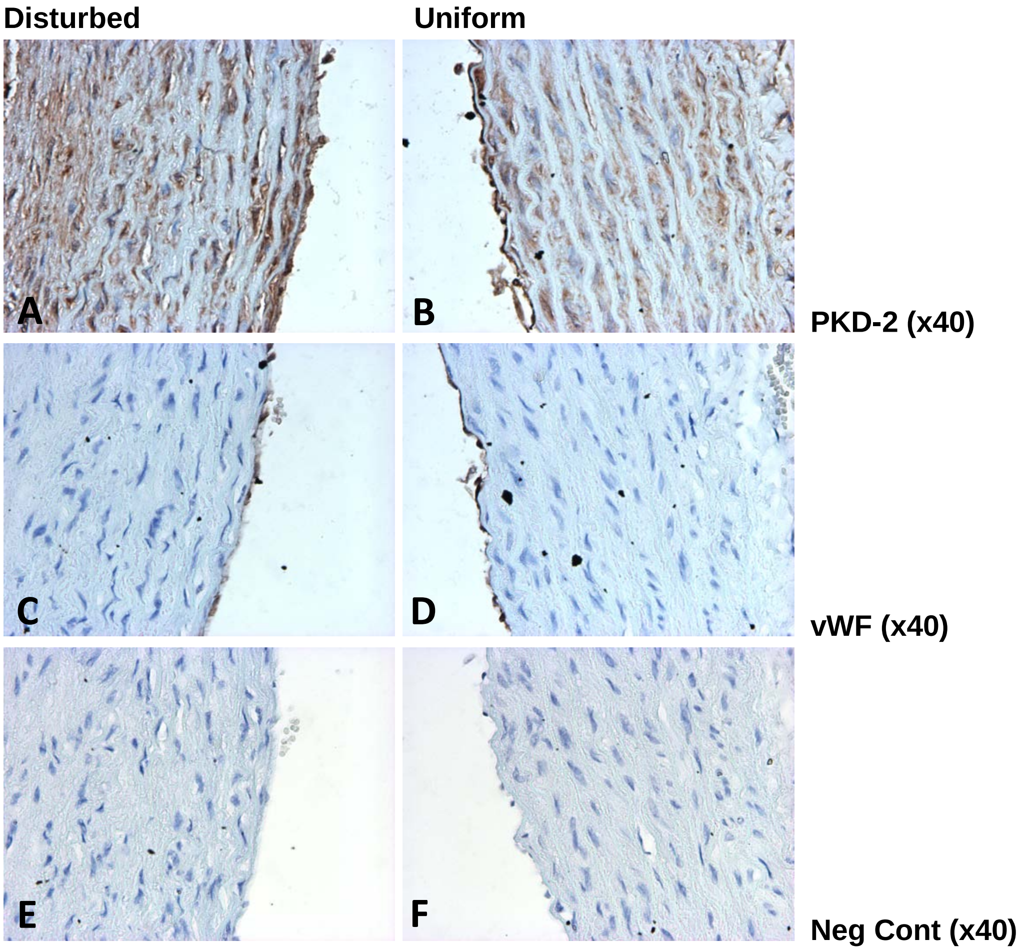

Supplementary Fig 2

Immunostaining of ZFP36 in disturbed (A) and uniform (B) flow pattern regions of rat aorta. C and D, vWF staining in disturbed and uniform plow pattern regions, respectively; E and F, negative control.

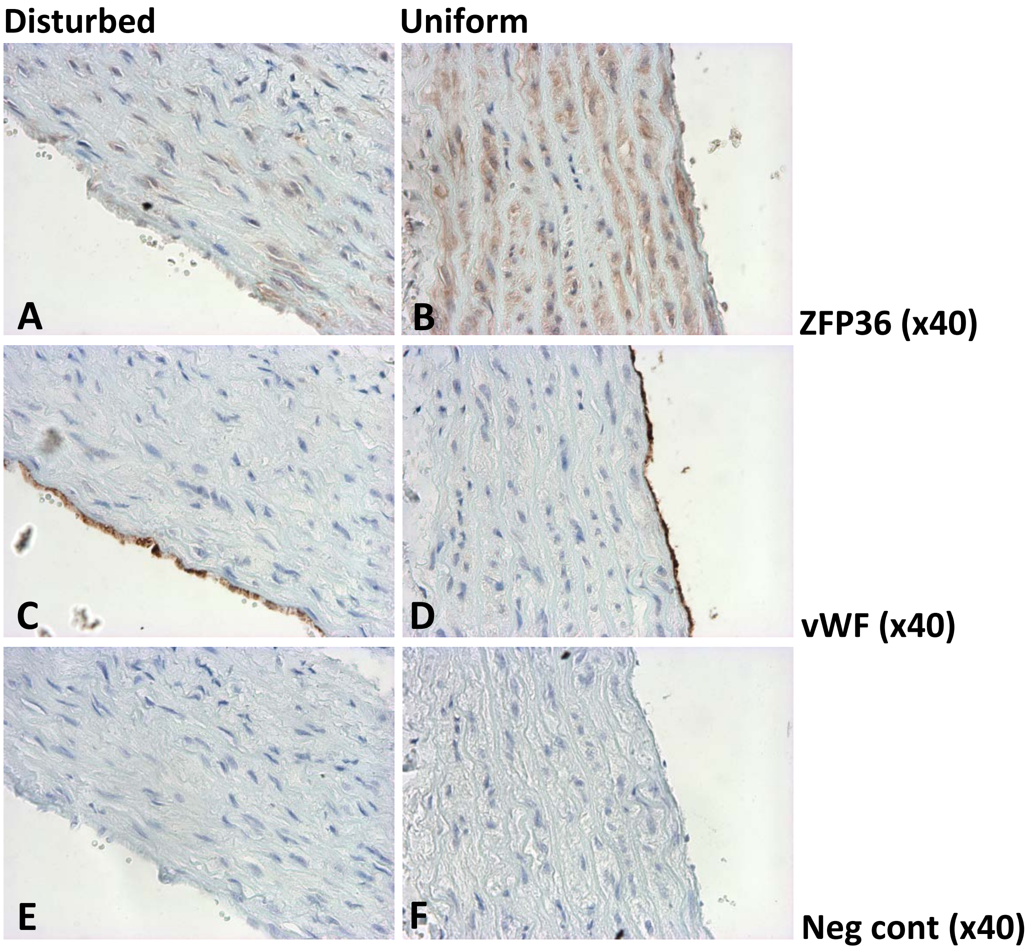

Supplementary Table 1A: List of genes selected using expression screening (for detailed description of selection see materials and methods and result section). Arbitrary cut off of >0.50 was chosen for expression probability in Human dataset. **(RED*)** Query genes.

Gene Symbol	Expression Probability	Name
KLF2*	0.87	Kruppel-like factor 2 (lung)
DUSP1	0.84	Dual specificity phosphatase 5
LATS2	0.84	Large tumor suppressor, homolog 2 (Drosophila)
THBD*	0.83	Thrombomodulin
ZFP36	0.83	Zinc finger protein 36
CD93	0.81	CD93 molecule
MCL1	0.79	Myeloid cell leukemia sequence 1 (BCL2-related)
EMP1	0.78	Epithelial membrane protein 1
MYADM	0.76	Myeloid-associated differentiation marker
JUNB	0.76	Jun B proto-oncogene
SERTAD1	0.75	SERTA domain containing 1
GADD45B	0.74	Growth arrest and DNA-damage-inducible, beta
FOS	0.73	C-FOS/AP-1 FBJ murine osteosarcoma viral oncogene homolog
EPAS1	0.73	Endothelial PAS domain protein 1
KLF4*	0.72	Kruppel-like factor 4 (gut)
CRISPLD2	0.72	Cysteine-rich secretory protein LCCL domain containing 2
TGFBR2	0.71	Transforming growth factor, beta receptor II (70/80kDa)
ENG	0.70	Endoglin
CDKN1A	0.70	Cyclin-dependent kinase inhibitor 1A /p21
CYR61	0.69	Cysteine-rich, angiogenic inducer, 61
MAFF	0.69	V-maf musculoaponeurotic fibrosarcoma oncogene homolog F (avian)
HEG1	0.68	HEG homolog 1 (zebrafish)
PECAM1	0.68	Platelet/endothelial cell adhesion molecule
SOCS3	0.68	Suppressor of cytokine signaling 3
IL1R1	0.68	Interleukin 1 receptor, type I
ADAMTS1	0.68	ADAM metallopeptidase with thrombospondin type 1 motif, 1
HBEGF	0.68	Heparin-binding EGF-like growth factor
SH2B3	0.68	SH2B adaptor protein 3
SLC2A3	0.67	Solute carrier family 2
LOC387763	0.67	Chromosome 11 open reading frame 96
CEBPD	0.66	CCAAT/enhancer binding protein (C/EBP), delta
IGFBP7	0.66	Insulin-like growth factor binding protein 7
THBS1	0.66	Thrombospondin 1
PTPRE	0.66	Protein tyrosine phosphatase, receptor type, E
STOM	0.66	Stomatin
DSE	0.65	Dermatan sulfate epimerase
TIMP1	0.65	TIMP metallopeptidase inhibitor 1
RHOB	0.64	Ras homolog gene family, member B
SGK1	0.64	Serum- and Glucocorticoid-Inducible Kinase 1
ATF3	0.64	Activating transcription factor 3

EGR1	0.64	Early growth response 1
NID1	0.63	Nidogen 1
LAMC1	0.63	Laminin, gamma 1
KLF10	0.63	Kruppel-like factor 10
TAGLN	0.63	Transgelin
ITGA5	0.63	Integrin, alpha 5
KIAA1754	0.63	Inositol 1,4,5-trisphosphate receptor interacting protein
KIAA0247	0.63	KIAA0247
CHSY1	0.62	Chondroitin sulfate synthase 1
PICALM	0.62	Phosphatidylinositol binding clathrin assembly protein
CAV1	0.62	CAV1 caveolin 1
CSGALNACT2	0.62	Chondroitin sulfate N-acetylgalactosaminyltransferase 2
KIAA1949	0.62	KIAA1949
METRNL	0.62	Meteorin
ELTD1	0.61	EGF, latrophilin and seven transmembrane domain containing 1
TIMP3	0.61	TIMP metallopeptidase inhibitor 3
KLF6	0.61	Kruppel-like factor 6
BTG2	0.61	BTG family, member 2
SWAP70	0.61	Switching B-cell complex 70kDa subunit
IER2	0.61	Immediate early response 2
CDH5	0.60	Cadherin 5, type 2
PTRF	0.60	Polymerase I and transcript release factor
CTGF	0.60	Connective tissue growth factor
DAB2	0.60	Disabled homolog 2, mitogen-responsive phosphoprotein
SNF1LK	0.60	SNF1-like kinase
TIE1*	0.59	Tyrosine kinase with immunoglobulin-like and EGF-like domains 1
ZCCHC24	0.59	Zinc finger, CCHC domain containing 24
PMP22	0.59	Peripheral myelin protein 22
KCTD12	0.59	Potassium channel tetramerisation domain containing 12
GNG11	0.59	Guanine nucleotide binding protein (G protein), gamma 11
ZFP36L1	0.59	Zinc finger protein 36, C3H type-like 1
TRAM2	0.59	Translocation associated membrane protein 2
TUBB6	0.58	Tubulin, beta 6
PPP1R15A	0.58	Protein phosphatase 1, regulatory (inhibitor) subunit 15A
PLEKHO2	0.58	Pleckstrin homology domain containing, family O member 2
HLX	0.57	H2.0-like homeobox
CEBPB	0.57	CCAAT/enhancer binding protein (C/EBP), beta
FOSB	0.57	FBJ murine osteosarcoma viral oncogene homolog B
SNRK	0.56	SNF related kinase
DUSP5	0.56	Dual specificity phosphatase 5
RGS2	0.56	Regulator of G-protein signaling 2, 24kDa
ERG	0.56	V-ets erythroblastosis virus E26 oncogene
PKD2*	0.55	Polycystic kidney disease 2
PPAP2B	0.55	Phosphatidic acid phosphatase type 2B
EHD2	0.55	EH-domain containing 2

CRTC3	0.55	CREB regulated transcription coactivator 3
COL6A3	0.55	Collagen, type VI, alpha 3
FERMT2	0.55	Fermitin family member 2
FILIP1L	0.54	Filamin A interacting protein 1-like
LHFP	0.54	Lipoma HMGIC fusion partner
NRP1	0.54	Neuropilin 1
LAMA4	0.54	Laminin, alpha 4 [
FLI1	0.54	Friend leukemia virus integration 1
PEA15	0.54	Phosphoprotein enriched in astrocytes 15
TGFB1I1	0.54	Transforming growth factor beta 1 induced transcript 1
ZEB2	0.54	Zinc finger E-box binding homeobox 2
NR4A2	0.54	Nuclear receptor subfamily 4, group A,
GRK5	0.53	G protein-coupled receptor kinase 5
VWF	0.53	Von Willebrand factor
MSN	0.53	Moesin
ARRDC3	0.53	Arrestin domain containing 3
ANXA5	0.53	Annexin A5
RAB31	0.53	Member RAS oncogene family
GEM	0.53	GTP binding protein
NFIL3	0.53	Nuclear factor, interleukin 3 regulated
FAM26B	0.52	Family with sequence similarity 26
ETS2	0.52	V-ets erythroblastosis virus E26 oncogene homolog 2 (avian)
CXCR7	0.52	Chemokine (C-X-C motif) receptor 7
IL4R	0.52	Interleukin 4 receptor
MAP3K8	0.52	Mitogen-activated protein kinase kinase kinase 8
PTGER4	0.52	Prostaglandin E receptor 4
NR4A1	0.52	Nuclear receptor subfamily 4, group A, member 1
ESAM	0.51	Endothelial cell adhesion molecule
GPR116	0.51	G protein-coupled receptor 116
RAB6IP1	0.51	DENN/MADD domain containing 5A
COL4A1	0.51	Collagen, type IV, alpha 1
KLF9	0.51	Kruppel-like factor 9
CALD1	0.51	Caldesmon 1
ANXA1	0.51	Annexin A1
PHLDB2	0.51	Pleckstrin homology-like domain, family B, member 2
CCL2	0.51	Chemokine (C-C motif) ligand 2
SNX9	0.51	Sorting nexin 9

Supplementary Table 1B: Expression screening using 5 and 9 query genes, respectively (see the Result section). Yellow marks the position of extra genes appearing in the selection with 5 query genes. #; query genes.

9 queries	**5 Queries**	**9 queries**	**5 Queries**	**9 queries**	**5 Queries**
KLF2#	***KLF2#***	*NID1*	*NID1*	***PKD2#***	***PKD2#***
DUSP1	*DUSP1*	*LAMC1*	*LAMC1*	*PPAP2B*	*PPAP2B*
LATS2	*LATS2*	*KLF10*	*KLF10*	*EHD2*	*EHD2*
THBD#	***THBD#***	*TAGLN*	*TAGLN*	*CRTC3*	*CRTC3*
ZFP36	*ZFP36*	*ITGA5*	*ITGA5*	*COL6A3*	*COL6A3*
CD93	*CD93*	---	*KIAA1754*	*FERMT2*	*FERMT2*
MCL1	*MCL1*	*KIAA0247*	*KIAA0247*	*FILIP1L*	*FILIP1L*
EMP1	*EMP1*	*CHSY1*	*CHSY1*	*LHFP*	*LHFP*
MYADM	*MYADM*	*PICALM*	*PICALM*	*NRP1*	*NRP1*
JUNB	*JUNB*	*CAV1*	*CAV1*	*LAMA4*	*LAMA4*
SERTAD1	*SERTAD1*	*CSGALNACT2*	*CSGALNACT2*	*FLI1*	*FLI1*
GADD45B	*GADD45B*	*KIAA1949*	*KIAA1949*	*PEA15*	*PEA15*
FOS	*FOS*	*METRNL*	*METRNL*	*TGFB1I1*	*TGFB1I1*
EPAS1	*EPAS1*	*ELTD1*	*ELTD1*	*ZEB2*	*ZEB2*
KLF4#	***KLF4#***	*TIMP3*	*TIMP3*	*NR4A2*	*NR4A2*
CRISPLD2	*CRISPLD2*	*KLF6*	*KLF6*	*GRK5*	*GRK5*
TGFBR2	*TGFBR2*	*BTG2*	*BTG2*	*VWF*	*VWF*
ENG	*ENG*	*SWAP70*	*SWAP70*	*MSN*	*MSN*
CDKN1A	*CDKN1A*	*IER2*	*IER2*	*ARRDC3*	*ARRDC3*
CYR61	*CYR61*	*CDH5*	*CDH5*	*ANXA5*	*ANXA5*
MAFF	*MAFF*	*PTRF*	*PTRF*	*RAB31*	*RAB31*
HEG1	*HEG1*	*CTGF*	*CTGF*	*GEM*	*GEM*
PECAM1	*PECAM1*	*DAB2*	*DAB2*	*NFIL3*	*NFIL3*
SOCS3	*SOCS3*	---	*SNF1LK*	---	*FAM26B*
IL1R1	*IL1R1*	***TIE1#***	***TIE1#***	*ETS2*	*ETS2*
ADAMTS1	*ADAMTS1*	*ZCCHC24*	*ZCCHC24*	*CXCR7*	*CXCR7*
HBEGF	*HBEGF*	*PMP22*	*PMP22*	*IL4R*	*IL4R*
SH2B3	*SH2B3*	*KCTD12*	*KCTD12*	*MAP3K8*	*MAP3K8*
SLC2A3	*SLC2A3*	*GNG11*	*GNG11*	*PTGER4*	*PTGER4*
---	*LOC387763*	*ZFP36L1*	*ZFP36L1*	*NR4A1*	*NR4A1*
CEBPD	*CEBPD*	*TRAM2*	*TRAM2*	*ESAM*	*ESAM*
IGFBP7	*IGFBP7*	*TUBB6*	*TUBB6*	*GPR116*	*GPR116*
THBS1	*THBS1*	*PPP1R15A*	*PPP1R15A*	---	*RAB6IP1*
PTPRE	*PTPRE*	*PLEKHO2*	*PLEKHO2*	*COL4A1*	*COL4A1*
STOM	*STOM*	*HLX*	*HLX*	*KLF9*	*KLF9*
DSE	*DSE*	*CEBPB*	*CEBPB*	*CALD1*	*CALD1*
TIMP1	*TIMP1*	*FOSB*	*FOSB*	*ANXA1*	*ANXA1*
RHOB	*RHOB*	*SNRK*	*SNRK*	*PHLDB2*	*PHLDB2*
SGK1	*SGK1*	*DUSP5*	*DUSP5*	*CCL2*	*CCL2*
ATF3	*ATF3*	*RGS2*	*RGS2*	*SNX9*	*SNX9*
EGR1	*EGR1*	*ERG*	*ERG*		

Supplementary Table 2: Analysis steps 2 and 3 using data from the ASAP database on genes selected using expression screening. ASAP microarray data set was analyzed using multivariate analysis (for detailed description of selection see materials and methods and result section). ***(a)*** Thoracic aorta, ***(b)*** Mammary artery. #, Query genes; **red** indicates significant correlation with one or more query gene (R>0.40) as well as differential expression following FDR 5%; **NA** Probe-set for gene missing. **Correlation coefficients:** Pink boxes indicate significant inverse correlations; blue boxes indicate significant positive correlations.

(*a*) Thoracic Aorta

Gene Symbol	KLF2	KLF4	PKD2	THBD	TIE1	Cuspidity
KLF2#	1	0.6	-0.31	0.31	0.26	0.0105
DUSP1	0.12	0.42	0.26	0.18	-0.06	0.623
LATS2	0.33	0.15	0.36	0.37	-0.36	0.595
THBD#	0.31	0.44	-0.05	1	-0.01	0.246
ZFP36	0.38	0.69	-0.46	0.51	0.3	2.22E-05
CD93	0.2	0.41	-0.31	0.68	0.17	0.000794
MCL1	-0.14	0.22	0.34	0.25	-0.12	0.451
EMP1	0.13	0.39	-0.28	0.32	0.38	0.00106
MYADM	-0.05	0.14	0.2	0.53	-0.19	0.501
JUNB	0.47	0.66	-0.32	0.46	0.13	2.60E-05
SERTAD1	0.16	0.33	0.03	0.24	-0.09	0.358
GADD45B	0.14	0.39	-0.21	0.25	0.11	0.0327
FOS	0.43	0.75	-0.31	0.26	0.27	6.31E-05
EPAS1	-0.08	0.1	0.24	0.2	-0.19	0.395
KLF4#	0.6	1	-0.36	0.44	0.35	8.29E-05
CRISPLD2	0.26	0.52	-0.48	0.06	0.62	0.014
TGFBR2	0.02	0.04	0.33	0.41	-0.17	0.321
ENG	0.34	0.32	-0.32	0.48	0.15	0.000117
CDKN1A	0.27	0.5	-0.27	0.23	0.04	0.002
CYR61	0.14	0.1	0.26	0.16	-0.06	0.314
MAFF	0.48	0.3	0.09	0.34	-0.05	0.947
HEG1	-0.22	0.12	0.15	0.39	-0.01	0.853
PECAM1	0.26	0.51	-0.4	0.17	0.5	0.000899
SOCS3	0.49	0.7	-0.6	0.24	0.5	2.84E-07
IL1R1	0.2	0.34	-0.11	0.42	0.02	0.0104
ADAMTS1	0.2	0.22	0.3	0.43	-0.06	0.75
HBEGF	0.1	0.34	-0.08	-0.09	0.35	0.0399
SH2B3	0.6	0.5	-0.57	0.26	0.42	8.03E-07
SLC2A3	0.37	0.66	-0.49	0.39	0.42	6.51E-08
LOC387763						NA
CEBPD	0.58	0.63	-0.44	0.22	0.26	0.000296
IGFBP7	-0.08	-0.1	0.38	0.28	-0.4	0.0438
THBS1	0.21	0.16	-0.14	0.48	0.01	0.00201
PTPRE	0.29	0.5	-0.53	0.29	0.25	4.96E-07

STOM	0.19	0.33	0.07	0.29	0.08	0.153
DSE	-0.05	0.22	-0.1	0.1	0.12	0.00489
TIMP1	0.42	0.27	-0.3	0.36	0.08	0.00329
RHOB	0.04	0.27	0.27	0.14	0.03	0.315
SGK1	0.16	0.47	-0.31	0.29	0.2	0.000461
ATF3	0.21	0.45	-0.04	-0.04	0.18	0.0618
EGR1	0.25	0.57	-0.19	0.5	0.1	0.000777
NID1	-0.01	0.28	-0.16	0.47	0.13	0.00197
LAMC1	-0.33	-0	0.5	0.24	-0.28	0.0354
KLF10	-0.02	0.23	0.09	0.31	-0.21	0.597
TAGLN	0.06	-0.1	0.47	0.05	-0.57	0.0593
ITGA5	0.27	0.37	-0.28	0.47	0.12	9.00E-04
KIAA1754						NA
KIAA0247	0.32	0.39	-0.28	0.51	0.09	5.25E-05
CHSY1	0.4	0.3	-0.16	0.24	0.09	0.124
PICALM	-0.09	0.01	0.23	0.31	-0.09	0.991
CAV1	-0.03	0	0.4	0.03	-0.25	0.0491
CSGALNACT2	-0.06	0.06	0.35	0.02	-0.26	0.859
KIAA1949	0.39	0.47	-0.18	0.36	-0.04	0.107
METRNL	0.15	0.11	-0.22	-0.11	0.08	0.383
ELTD1	0.27	0.52	-0.51	0.16	0.68	3.03E-05
TIMP3	-0.08	0.18	0.03	-0.08	0.1	0.22
KLF6	0.07	0.13	0.26	0.19	-0.11	0.494
BTG2	0.33	0.77	-0.35	0.22	0.35	1.35E-05
SWAP70	0.03	0.15	0.37	0.31	-0.35	0.125
IER2	0.31	0.63	-0.23	0.48	0.26	0.000429
CDH5	0.42	0.47	-0.54	0.22	0.7	2.52E-05
PTRF	0.28	0.2	0.23	0.39	-0.41	0.594
CTGF	-0.02	-0.2	0.49	0.33	-0.25	0.0154
DAB2	0.08	0.42	-0.21	0.44	0.15	0.000298
SNF1LK						NA
TIE1[#]	0.26	0.35	-0.51	-0.01	1	0.0333
ZCCHC24	0.37	0.34	-0.16	0.2	0.03	0.0879
PMP22	-0.09	0.2	-0.09	0.27	0.23	0.101
KCTD12	0.16	0.25	-0.13	0.05	0.19	0.193
GNG11	0.15	0.28	-0.01	-0.08	0.06	0.993
ZFP36L1	0.28	0.48	-0.25	0.47	0.11	0.0101
TRAM2	-0.09	0.17	0.21	0.4	-0.24	0.65
TUBB6	0.24	0.19	0.2	0.11	-0.25	0.324
PPP1R15A	0.45	0.49	0.11	0.37	-0.2	0.676
PLEKHO2	0.31	0.55	-0.58	0.37	0.41	6.75E-08
HLX	0.56	0.28	-0.52	-0.01	0.57	0.024
CEBPB	0.39	0.43	-0.36	0.15	0.05	0.000551
FOSB	0.51	0.74	-0.39	0.15	0.35	1.23E-05
SNRK	0.24	0.49	-0.02	0.27	0.26	0.0247
DUSP5	0.64	0.52	-0.49	0.05	0.52	8.17E-05

RGS2	0.23	0.17	-0.01	0.24	-0.06	0.11
ERG	0.2	0	0.51	0.35	-0.45	0.00718
PKD2[#]	-0.31	-0.4	1	-0.05	-0.51	7.03E-07
PPAP2B	-0.11	0.07	0.21	0.12	-0.06	0.946
EHD2	0.21	0.29	-0.03	0.54	-0.16	0.502
CRTC3	0.08	0.18	0.22	0.29	-0.28	0.287
COL6A3	0.02	0.36	-0.32	0.44	0.14	9.00E-05
FERMT2	-0.18	-0.2	0.77	-0.05	-0.47	0.000179
FILIP1L	0.1	0	0.25	-0.11	-0.18	0.146
LHFP	0.03	0.07	0.24	0.18	-0.19	0.16
NRP1	0.14	0.37	-0.37	0.33	0.25	0.000259
LAMA4	0.06	0.35	-0.24	0.17	0.27	0.0324
FLI1	0.25	0.46	-0.5	0.22	0.4	3.66E-06
PEA15	-0.05	0.09	0.36	0.02	-0.17	0.132
TGFB1I1	0.1	0.03	0.41	0.42	-0.38	0.0725
ZEB2	-0.03	0.29	-0.01	0.25	0.01	0.0274
NR4A2	0.31	0.74	-0.17	0.32	0.26	0.057
GRK5	-0.2	-0.2	0.71	0.04	-0.49	8.30E-05
VWF	0.12	0.3	-0.19	0.23	0.54	0.739
MSN	0.2	0.27	0.2	0.63	-0.22	0.985
ARRDC3	-0.04	0.26	-0.07	0.02	0.07	0.0465
ANXA5	-0.28	0	0.46	0.07	-0.29	0.184
RAB31	0.09	0.12	0.04	0.16	-0.09	0.0848
GEM	-0.22	-0.3	0.74	-0.07	-0.51	0.000193
NFIL3	0.13	0.35	0.1	0.01	0.11	0.869
FAM26B						NA
ETS2	0.25	0.17	0.34	0.53	-0.29	0.366
CXCR7	-0.31	0.04	0.21	-0.11	0.16	0.068
IL4R	0.29	0.46	-0.43	0.29	0.23	1.23E-06
MAP3K8	0.25	0.41	-0.12	0.25	0.07	0.0152
PTGER4	0.31	0.42	-0.53	0.21	0.45	1.92E-06
NR4A1	0.42	0.77	-0.22	0.36	0.23	0.0188
ESAM	0.06	0.21	-0.37	0.17	0.24	0.00209
GPR116	0.27	0.48	-0.55	0.26	0.55	1.99E-08
RAB6IP1						NA
COL4A1	-0.07	0.02	0.09	0.3	-0.16	0.976
KLF9	0.05	0.16	0.05	0.15	-0.05	0.259
CALD1	-0.09	0	0.6	0.31	-0.45	0.00108
ANXA1	0.28	0.11	0.21	0.21	-0.25	0.579
PHLDB2	-0.27	0.08	0.49	0.27	-0.39	0.764
CCL2	0.3	0.48	-0.5	0.28	0.23	1.32E-06
SNX9	0	0.17	0.45	0.42	-0.39	0.36

(b) Mammary Artery

Gene Symbol	KLF2	KLF4	PKD2	THBD	TIE1	Cuspidity
KLF2[#]	1	0.51	-0.53	0.22	0.29	0.622
DUSP1	0.27	0.43	0.22	0.14	0.02	0.843
LATS2	0.21	0.36	0.22	0.4	0.06	0.261
THBD[#]	0.22	0.32	-0.02	1	0.14	0.006
ZFP36	0.67	0.52	-0.4	0.32	0.36	0.915
CD93	0.03	0.22	0.08	0.73	0.05	0.102
MCL1	-0.37	0.11	0.63	0.04	-0.38	0.11
EMP1	0.07	0.45	-0.13	0.25	0.38	0.992
MYADM	0.29	0.32	0.08	0.58	0.22	0.821
JUNB	0.48	0.49	-0.09	0.13	0.09	0.109
SERTAD1	0.24	0.34	0.04	0.22	0.22	0.982
GADD45B	0.14	0.18	-0.1	0.03	0.28	0.742
FOS	0.5	0.58	-0.16	0.27	0.15	0.57
EPAS1	-0.14	0.05	0.11	0.46	0.18	0.306
KLF4[#]	0.51	1	-0.07	0.32	0.19	0.15
CRISPLD2	0.27	0.36	-0.41	0.12	0.45	0.0923
TGFBR2	-0.21	0.07	0.32	0.22	-0.17	0.677
ENG	0.4	0.25	0.01	0.42	0.01	0.31
CDKN1A	0.38	0.31	-0.05	0.31	0.07	0.623
CYR61	0.26	0.47	0.22	0.31	0.06	0.354
MAFF	0.43	0.3	0.06	0.22	0.06	0.32
HEG1	-0.29	0.16	0.34	0.47	0.11	0.939
PECAM1	-0.11	0.19	-0.04	0.1	0.53	0.497
SOCS3	0.58	0.27	-0.52	0.2	0.25	0.336
IL1R1	-0.18	0.32	0.4	0.27	-0.16	0.947
ADAMTS1	0.09	0.24	0.37	0.28	-0.22	0.196
HBEGF	0.17	0.32	0.02	0.09	0.33	0.71
SH2B3	0.68	0.27	-0.57	0.31	0.28	0.494
SLC2A3	0.18	0.41	0.02	0.22	0.01	0.438
LOC387763	NA			NA		NA
CEBPD	0.41	0.28	-0.56	0.02	0.38	0.217
IGFBP7	0.13	0.16	0.21	0.46	0	0.655
THBS1	-0.05	0.27	0.24	0.39	-0.02	0.00701
PTPRE	0.23	0.35	-0.15	0.5	0.22	0.204
STOM	-0.21	0.24	0.47	0.28	0.17	0.747
DSE	-0.32	0.23	0.52	0.14	-0.18	0.953
TIMP1	0.34	0.27	-0.01	0.48	-0.02	0.0178
RHOB	0.27	0.5	0.17	0.4	0.27	0.914
SGK1	0.03	0.23	0.32	0.09	-0.08	0.523
ATF3	0.16	0.35	0.2	0.15	0.01	0.549
EGR1	0.33	0.54	0.07	0.45	0.01	0.324

NID1	-0.11	0.07	-0.05	0.35	0.36	0.971
LAMC1	-0.31	0.1	0.51	0.42	-0.04	0.595
KLF10	-0.22	0.1	0.18	0.25	0.13	0.923
TAGLN	0.17	0.19	0.06	0.25	0.21	0.792
ITGA5	0.13	0.34	0.06	0.39	0.17	0.00164
KIAA1754	NA			NA		NA
KIAA0247	0.07	0.3	0.22	0.52	-0.03	0.0342
CHSY1	0.38	0.3	-0.09	0.12	0.16	0.375
PICALM	-0.44	-0.06	0.62	0.23	-0.26	0.701
CAV1	-0.35	-0.06	0.31	0.12	0.06	0.302
CSGALNACT2	-0.53	-0.03	0.63	-0.1	-0.37	0.968
KIAA1949	0.01	0.33	0.19	0.19	0	0.425
METRNL	0.53	0.37	-0.32	0.26	0.36	0.949
ELTD1	0.07	0.31	-0.13	0.02	0.17	0.624
TIMP3	0.04	0.25	-0.1	0.29	0.4	0.00325
KLF6	0.19	0.44	0.29	0.36	-0.03	0.303
BTG2	0.18	0.43	-0.03	0.07	0.26	0.158
SWAP70	-0.44	0	0.36	0.27	-0.15	0.633
IER2	0.59	0.54	-0.29	0.27	0.32	0.689
CDH5	0.32	0.32	-0.4	0.22	0.75	0.861
PTRF	0.23	0.28	0.01	0.55	0.18	0.478
CTGF	0.21	0.37	0.2	0.43	0	0.0766
DAB2	-0.09	0.31	0.25	0.3	-0.11	0.346
SNF1LK	NA		NA			NA
TIE1*	0.29	0.19	-0.4	0.14	1	0.338
ZCCHC24	0.3	0.46	-0.2	0.35	0.34	0.273
PMP22	0.06	0.35	-0.07	0.3	0.33	0.242
KCTD12	0.24	0.47	0.06	0.21	0.13	0.364
GNG11	-0.13	0.13	0.15	-0.02	-0.03	0.0833
ZFP36L1	-0.12	0.12	0.07	0.35	0.06	0.0636
TRAM2	-0.27	0.12	0.44	0.29	-0.01	0.688
TUBB6	0.33	0.34	-0.01	0.37	0.24	0.824
PPP1R15A	0.28	0.41	0.17	0.3	0.02	0.478
PLEKHO2	0.41	0.46	-0.31	0.4	0.35	0.985
HLX	0.56	0.16	-0.66	-0.03	0.54	0.0377
CEBPB	0.48	0.28	-0.45	0.16	0.42	0.353
FOSB	0.56	0.48	-0.32	0.03	0.23	0.664
SNRK	-0.43	0.1	0.39	0.05	0.06	0.203
DUSP5	0.69	0.17	-0.59	-0.01	0.41	0.111
RGS2	-0.3	-0.23	0.18	-0.1	-0.4	0.909
ERG	-0.18	0.02	0.5	0.1	-0.47	0.0332
PKD2[#]	-0.53	-0.07	1	-0.02	-0.4	0.113
PPAP2B	-0.04	0.22	0.05	0.33	0.29	0.104
EHD2	0.38	0.3	-0.16	0.6	0.28	0.873
CRTC3	0.03	0.17	0.13	0.48	0.13	0.597
COL6A3	0.04	0.38	0.09	0.26	0.06	0.243

FERMT2	-0.56	-0.08	0.66	0.03	-0.23	0.595
FILIP1L	0.01	0.01	0.03	0.01	-0.04	0.0821
LHFP	0.03	0.32	0.18	0.3	0.09	0.277
NRP1	-0.07	0.26	0.14	0.44	0.11	0.592
LAMA4	-0.28	0.17	0.21	0.26	-0.01	0.57
FLI1	0.23	0.48	-0.05	0.28	0.16	0.399
PEA15	-0.09	0.17	0.41	0.13	-0.13	0.574
TGFB1I1	0.37	0.3	-0.09	0.55	0.3	0.699
ZEB2	-0.46	-0.06	0.31	0.22	-0.07	0.537
NR4A2	0.39	0.33	-0.23	-0.14	0.23	0.23
GRK5	-0.33	0.04	0.53	0.29	-0.13	0.469
VWF	0.18	0.27	-0.18	0.4	0.61	0.0811
MSN	-0.01	0.37	0.37	0.46	-0.04	0.191
ARRDC3	-0.52	-0.11	0.22	-0.18	-0.21	0.178
ANXA5	-0.3	0.17	0.63	0.17	-0.21	0.973
RAB31	-0.28	0.11	0.55	0.28	-0.32	0.469
GEM	-0.19	0.13	0.65	0.11	-0.17	0.475
NFIL3	-0.36	0.03	0.38	-0.03	-0.05	0.0618
FAM26B	NA		NA			NA
ETS2	0.09	0.2	0.32	0.31	-0.11	0.775
CXCR7	0.03	0.34	0.2	0.15	0.05	0.612
IL4R	-0.03	0.23	0.13	0.59	0.12	0.226
MAP3K8	-0.03	0.37	0.39	0.17	-0.2	0.104
PTGER4	0.33	0.23	-0.15	0.37	0.25	0.271
NR4A1	0.6	0.55	-0.25	0.16	0.31	0.813
ESAM	0.06	0.11	0	0.5	0.06	0.244
GPR116	0.01	0.1	-0.25	0.17	0.04	0.722
RAB6IP1	NA		NA			NA
COL4A1	-0.03	0.16	0.25	0.5	0.13	0.256
KLF9	0.42	0.36	-0.18	0.32	0.28	0.59
CALD1	-0.54	-0.12	0.64	0.28	-0.38	0.478
ANXA1	-0.31	0.03	0.45	0.11	-0.4	0.223
PHLDB2	-0.62	-0.19	0.56	0.14	-0.36	0.326
CCL2	0.07	0.25	-0.05	0.33	-0.12	0.436
SNX9	-0.37	0.11	0.64	0.35	-0.27	0.707

Supplementary table 3: Genes differentially expressed between BAV and TAV (same as table 1 but with references)

Gene	Flow	Function(s) and properties
ZFP36L1	X[*4] (6)	Zinc finger protein, modulation and destabilization VEGF mRNA (7), **wound healing** (8)
ZFP36	X[*4] (6)	Modulation and destabilisation VEGF mRNA (9), **wound healing** (10)
FLI1	X (11)	**Angiogenesis**, maturation and stabilization of vessels (12), **wound healing** (13)
EGR1	X (14)	Regulates angiopoietin-1 induced EC migration and proliferation (15), **wound healing** (10)
IER2	X[*4] (6)	Mediates EGF-dependent left-right asymmetry patterning in zebrafish (16)
SLC2A3	X[*4] (6)	Involved in tumor **angiogenesis** (17)
FOSB	X (18)	Induced by mechanical stress and cardiac ischemia (19) and VEGF (20), **wound healing** (21)
TIMP1	X (22)	**Anti-angiogenesis** (23), **wound healing** (24)
GRK5	X (25)	Regulation of vasoconstriction in VSMC (26), histone deacetylase (HDAC) kinase in cardiomyocytes (27)
CCL2	X (28)	Involved in neovascularization and **angiogenesis** (29), **wound healing** (30)
NID1	X (31)	Ingredient of vascular basement membranes (32), **wound healing** (33)
ITGA5	X (34)	Fibronectin receptor (35)
SOCS3	X (36)	Negative regulator of cytokines (37), **wound healing** (38)
FOS	X (14)	Regulation of VEGF, **angiogenesis** (39), **wound healing** (21)
KLF2[#]	X (40)	Regulates VEGFA, **angiogenesis** (41)
BTG2	X (36)	Anti-proliferative transcription factor (42), involved in modulation of VEGF regulated **wound repair** (43)
SGK1	X (44)	Involved in vascular remodelling during **angiogenesis** (45), **wound healing** (46)
PLEKHO2	?	No functional studies performed according to PubMed
PTGER4	X (36)	Mediates Prostaglandin E2 stimulated **VEGF** expression, **angiogenesis** (47), **wound healing** (48)
COL6A3	X*[2] (49)	ECM component, regulated by TGFβ (50)
FERMT2	?	**Wound healing** (51), regulated by TGF-β1 (52), **angiogenesis** (53)
IL1R1	X (44)	**Wound healing** (54)
PKD2[#]	X (55)	Ca^{2+}- permeable channel of cilia (55), embryonic left-right asymmetry (56), **wound healing** (57)

GEM	X (58)	Regulation of voltage-gated Ca^{2+}- channel (59)
DAB2	X*[2] (49)	Embryonic **angiogenesis** via VEGF induction (60),TGFβ stimulated fibronectin synthesis in **wound healing** (61)
GPR116	X*[2] (49)	Specifically expressed in the microvasculature (62)
DUSP5	X (63)	strongly induced by VEGF in EC (64), EC specific in vessels development (65)
CEBPB	?	It interacts directly with subunits of NF*k*B to augment gene expression by FOS multimeric transcription factors (66)
KLF4[#]	X (6)	Modulates phenotype of VSMCs (67), **angiogenesis** (68), **wound healing** (69)
SH2B3	?	Stabilization of thrombi within vessels (70), regulates EPC kinetics in vascular regeneration (71)
JUNB	X (36)	Regulates VEGF expression in an NFkB dependent manner (72), **angiogenesis**, **wound healing** (21)
CDH5	X (73)	Ingredient of shear stress sensor complex, **angiogenesis** (74), EC specific in vessels development (65)
CD93	X*[2] (49)	Vascular remodelling and **angiogenesis** (75)
CDKN1A	X (34)	Involved in regulation of EC senescence and permeability (76), **wound healing** (77)
PTPRE	X (25)	Negative regulation of EC proliferation, possible role in **angiogenesis** (78)
CALD1	X*[2] (49)	Cytoskeletal protein **anti-angiogenesis** (79), implicated in the migration of EPC (80)
ERG	X*[2] (81)	A**ngiogenesis** (12)**, wound healing** through regulation of ECM (82)
ELTD1	X[*4] (6)	Specifically expressed in the microvasculature (62)
ENG	X (83)	Vascular TGFβ co-receptor, **angiogenesis** (84), **wound healing** (85)
PECAM1	X (73)	Ingredient of shear stress sensor complex**, Angiogenesis** (86), **wound healing** (87)
CEBPD	X*[4] (6)	It interacts directly with subunits of NF*k*B to augment gene expression by FOS multimeic transcription factors (66)
THBS1	X (34)	**Anti-angiogenesis** (88), **wound healing** (89)
KIAA0247	?	Regulator of cell cycle (90)
IL4R	X (36)	IL4 receptor, IL4 has been indicated in both promoting (91) and blocking (92) **angiogenesis**

(**X**) Induced by shear stress according to the literature; **(X*[2])** by KLF2 or**(X*[4])** KLF4 over-expression (extracted from literature search); **(?)** Not cited in the literature. #; query genes.

Supplementary references

1. Folkersen L*, et al.* (2011) Unraveling the divergent gene expression profiles in bicuspid and tricuspid aortic valve patients with thoracic aortic dilatation - the ASAP study. *Mol Med*. 17:1365-73
2. Jackson V*, et al.* (2011) Bicuspid aortic valve leaflet morphology in relation to aortic root morphology: a study of 300 patients undergoing open-heart surgery. *Eur J Cardiothorac Surg.* 40:e118-24.
3. Nilsson R*, et al.* (2009) Discovery of genes essential for heme biosynthesis through large-scale gene expression analysis. *Cell Metab.* 10:119-30.
4. Baughman JM*, et al.* (2009) A computational screen for regulators of oxidative phosphorylation implicates SLIRP in mitochondrial RNA homeostasis. *PLoS Genet* 5:e1000590.
5. Irizarry RA*, et al.* (2003) Exploration, normalization, and summaries of high density oligonucleotide array probe level data. *Biostatistics* 4:249-64.
6. Villarreal G, Jr.*, et al.* (2010) Defining the regulation of KLF4 expression and its downstream transcriptional targets in vascular endothelial cells. *Biochem Biophys Res Commun.* 391:984-9.
7. Planel S, Salomon A, Jalinot P, Feige JJ, Cherradi N (2010) A novel concept in antiangiogenic and antitumoral therapy: multitarget destabilization of short-lived mRNAs by the zinc finger protein ZFP36L1. *Oncogene* 29:5989-6003.
8. Pastar I*, et al.* (2010) Attenuation of the Transforming Growth Factor beta-Signaling Pathway in Chronic Venous Ulcers. *Mol Med.* 16:92-101.
9. Suswam E*, et al.* (2008) Tristetraprolin down-regulates interleukin-8 and vascular endothelial growth factor in malignant glioma cells. *Cancer Res.* 68:674-82.
10. Dayem MA*, et al.* (2003) Early gene expression in wounded human keratinocytes revealed by DNA microarray analysis. *Comp Funct Genomics* 4:47-55.
11. Yoshisue H*, et al.* (2002) Large scale isolation of non-uniform shear stress-responsive genes from cultured human endothelial cells through the preparation of a subtracted cDNA library. *Atherosclerosis* 162:323-34.
12. Sato Y (2001) Role of ETS family transcription factors in vascular development and angiogenesis. *Cell Struct Funct* 26:19-24.
13. Sakthianandeswaren A*, et al.* (2010) Fine Mapping of Leishmania major Susceptibility Locus lmr2 and Evidence of a Role for Fli1 in Disease and Wound Healing. *Infect Immun* 78:2734-44.
14. Nagel T, Resnick N, Dewey CF, Jr., Gimbrone MA, Jr. (1999) Vascular endothelial cells respond to spatial gradients in fluid shear stress by enhanced activation of transcription factors. *Arterioscler Thromb Vasc Biol.* 19:1825-34.
15. Abdel-Malak NA, Mofarrahi M, Mayaki D, Khachigian LM, Hussain SN (2009) Early growth response-1 regulates angiopoietin-1-induced endothelial cell proliferation, migration, and differentiation. *Arterioscler Thromb Vasc Biol.* 29:209-16.
16. Hong SK, Dawid IB (2009) FGF-dependent left-right asymmetry patterning in zebrafish is mediated by Ier2 and Fibp1. *Proc Natl Acad Sci U S A* 106:2230-5.
17. Tsukioka M*, et al.* (2007) Expression of glucose transporters in epithelial ovarian carcinoma: correlation with clinical characteristics and tumor angiogenesis. *Oncol Rep.* 18:361-7.
18. Inoue D, Kido S, Matsumoto T (2004) Transcriptional induction of FosB/DeltaFosB gene by mechanical stress in osteoblasts. *J Biol Chem.* 279:49795-803.
19. Alfonso-Jaume MA*, et al.* (2006) Cardiac ischemia-reperfusion injury induces matrix metalloproteinase-2 expression through the AP-1 components FosB and JunB. *Am J Physiol Heart Circ Physiol.* 291:H1838-46.
20. Holmes DI, Zachary I (2004) Placental growth factor induces FosB and c-Fos gene expression via Flt-1 receptors. *FEBS Lett.* 557:93-8.

21. Shirai K, Okada Y, Saika S, Senba E, Ohnishi Y (2001) Expression of transcription factor AP-1 in rat lens epithelial cells during wound repair. *Exp Eye Res.* 73:461-8.
22. Milkiewicz M, Uchida C, Gee E, Fudalewski T, Haas TL (2008) Shear stress-induced Ets-1 modulates protease inhibitor expression in microvascular endothelial cells. *J Cell Physiol.* 217:502-10.
23. Reed MJ, Koike T, Sadoun E, Sage EH, Puolakkainen P (2003) Inhibition of TIMP1 enhances angiogenesis in vivo and cell migration in vitro. *Microvasc Res.* 65:9-17.
24. Stevenson TJ, Vinarsky V, Atkinson DL, Keating MT, Odelberg SJ (2006) Tissue inhibitor of metalloproteinase 1 regulates matrix metalloproteinase activity during newt limb regeneration. *Dev Dyn.* 235:606-16.
25. Conway DE, Williams MR, Eskin SG, McIntire LV (2010) Endothelial cell responses to atheroprone flow are driven by two separate flow components: low time-average shear stress and fluid flow reversal. *Am J Physiol.* 298:H367-74.
26. Morris GE*, et al.* (2011) G protein-coupled receptor kinase 2 and arrestin2 regulate arterial smooth muscle P2Y-purinoceptor signalling. *Cardiovasc Res.* 89:193-203.
27. Martini JS*, et al.* (2008) Uncovering G protein-coupled receptor kinase-5 as a histone deacetylase kinase in the nucleus of cardiomyocytes (vol 105, pg 12457, 2008). *Proc Natl Acad Sci U S A* 105:17206-06.
28. Shyy YJ, Hsieh HJ, Usami S, Chien S (1994) Fluid shear stress induces a biphasic response of human monocyte chemotactic protein 1 gene expression in vascular endothelium. *Proc Natl Acad Sci U S A* 91:4678-82.
29. Niu J, Kolattukudy PE (2009) Role of MCP-1 in cardiovascular disease: molecular mechanisms and clinical implications. *Clin Sci.* 117:95-109.
30. Yahiaoui L, Gvozdic D, Danialou G, Mack M, Petrof BJ (2008) CC family chemokines directly regulate myoblast responses to skeletal muscle injury. *J Physiol.* 586:3991-4004.
31. Chen BP*, et al.* (2001) DNA microarray analysis of gene expression in endothelial cells in response to 24-h shear stress. *Physiol Genomics* 7:55-63.
32. Hallmann R*, et al.* (2005) Expression and function of laminins in the embryonic and mature vasculature. *Physiol Rev.* 85:979-1000.
33. Baranowsky A*, et al.* (2010) Impaired wound healing in mice lacking the basement membrane protein nidogen 1. *Matrix Biol.* 29:15-21.
34. Chu TJ, Peters DG (2008) Serial analysis of the vascular endothelial transcriptome under static and shear stress conditions. *Physiol Genomics* 34:185-92.
35. Adkison LR*, et al.* (1994) The fibronectin receptor, alpha subunit (Itga5) maps to murine chromosome 15, distal to D15Mit16. *Mamm Genome* 5:456-7.
36. Shivaram GM*, et al.* (2010) Novel early response genes in osteoblasts exposed to dynamic fluid flow. *Philos Transact A Math Phys Eng Sci.* 368:605-16.
37. Yoshimura A (2009) Regulation of cytokine signaling by the SOCS and Spred family proteins. *Keio J Med.* 58:73-83.
38. Zhu BM*, et al.* (2008) SOCS3 negatively regulates the gp130-STAT3 pathway in mouse skin wound healing. *J Invest Dermatol.* 128:1821-9.
39. McColl BK, Stacker SA, Achen MG (2004) Molecular regulation of the VEGF family -- inducers of angiogenesis and lymphangiogenesis. *Apmis* 112:463-80.
40. Chiu JJ, Chien S (2011) Effects of disturbed flow on vascular endothelium: pathophysiological basis and clinical perspectives. *Physiol Rev.* 91:327-87.
41. Bhattacharya R*, et al.* (2005) Inhibition of vascular permeability factor/vascular endothelial growth factor-mediated angiogenesis by the Kruppel-like factor KLF2. *J Biol Chem.* 280:28848-51.
42. Winkler GS (2010) The mammalian anti-proliferative BTG/Tob protein family. *J Cell Physiol.* 222:66-72.
43. Bockhorn M*, et al.* (2007) VEGF is important for early liver regeneration after partial hepatectomy. *J Surg Res.* 138:291-9.

44. Parmar KM, *et al.* (2006) Integration of flow-dependent endothelial phenotypes by Kruppel-like factor 2. *J Clin Invest.* 116:49-58.
45. Catela C, Kratsios P, Hede M, Lang F, Rosenthal N (2010) Serum and glucocorticoid-inducible kinase 1 (SGK1) is necessary for vascular remodeling during angiogenesis. *Dev Dyn.* 239:2149-60.
46. Santini MP, *et al.* (2007) Enhancing repair of the mammalian heart. *Circ Res.* 100:1732-40.
47. Hatazawa R, *et al.* (2007) Prostaglandin E2 stimulates VEGF expression in primary rat gastric fibroblasts through EP4 receptors. *Inflammopharmacol.* 15:214-7.
48. Savla U, Appel HJ, Sporn PH, Waters CM (2001) Prostaglandin E(2) regulates wound closure in airway epithelium. *Am J Physiol Lung Cell Mol Physiol.* 280:L421-31.
49. Boon RA, *et al.* (2010) KLF2-induced actin shear fibers control both alignment to flow and JNK signaling in vascular endothelium. *Blood* 115:2533-42.
50. Verrecchia F, Chu ML, Mauviel A (2001) Identification of novel TGF-beta /Smad gene targets in dermal fibroblasts using a combined cDNA microarray/promoter transactivation approach. *J Biol Chem.* 276:17058-62.
51. He Y, Esser P, Schacht V, Bruckner-Tuderman L, Has C (2011) Role of kindlin-2 in fibroblast functions: implications for wound healing. *J Invest Dermatol.* 131:245-56.
52. Qu H, *et al.* (2011) Kindlin-2 regulates podocyte adhesion and fibronectin matrix deposition through interactions with phosphoinositides and integrins. *J Cell Sci.* 124:879-91.
53. Pluskota E, *et al.* (2011) The integrin coactivator kindlin-2 plays a critical role in angiogenesis in mice and zebrafish. *Blood* 117:4978-87.
54. Bujak M, *et al.* (2008) Interleukin-1 receptor type I signaling critically regulates infarct healing and cardiac remodeling. *Am J Pathol.* 173:57-67.
55. AbouAlaiwi WA, *et al.* (2009) Ciliary polycystin-2 is a mechanosensitive calcium channel involved in nitric oxide signaling cascades. *Circ Res.* 104:860-9.
56. Pennekamp P, *et al.* (2002) The ion channel polycystin-2 is required for left-right axis determination in mice. *Curr Biol.* 12:938-43.
57. Prasad S, McDaid JP, Tam FWK, Haylor JL, Ong ACM (2009) Pkd2 Dosage Influences Cellular Repair Responses following Ischemia-Reperfusion Injury. *Am J Pathol.* 175:1493-503.
58. Dai G, *et al.* (2004) Distinct endothelial phenotypes evoked by arterial waveforms derived from atherosclerosis-susceptible and -resistant regions of human vasculature. *Proc Natl Acad Sci U S A* 101:14871-6.
59. Beguin P, *et al.* (2005) 14-3-3 and calmodulin control subcellular distribution of Kir/Gem and its regulation of cell shape and calcium channel activity. *J Cell Sci.* 118:1923-34.
60. Cheong SM, Choi SC, Han JK (2006) Xenopus Dab2 is required for embryonic angiogenesis. *Bmc Dev Biol.* 6:63.
61. Hocevar BA, Prunier C, Howe PH (2005) Disabled-2 (Dab2) mediates transforming growth factor beta (TGFbeta)-stimulated fibronectin synthesis through TGFbeta-activated kinase 1 and activation of the JNK pathway. *J Biol Chem.* 280:25920-7.
62. Wallgard E, *et al.* (2008) Identification of a core set of 58 gene transcripts with broad and specific expression in the microvasculature. *Arterioscler Thromb Vasc Biol.* 28:1469-76.
63. Glossop JR, Cartmell SH (2009) Effect of fluid flow-induced shear stress on human mesenchymal stem cells: differential gene expression of IL1B and MAP3K8 in MAPK signaling. *Gene Expr Patterns* 9:381-8.
64. Bellou S, *et al.* (2009) VEGF autoregulates its proliferative and migratory ERK1/2 and p38 cascades by enhancing the expression of DUSP1 and DUSP5 phosphatases in endothelial cells. *Am J Physiol Cell Physiol.* 297:C1477-89.
65. Sumanas S, Jorniak T, Lin S (2005) Identification of novel vascular endothelial-specific genes by the microarray analysis of the zebrafish cloche mutants. *Blood* 106:534-41.
66. Stein B, Cogswell PC, Baldwin AS, Jr. (1993) Functional and physical associations between NF-kappa B and C/EBP family members: a Rel domain-bZIP interaction. *Mol Cell Biol.* 13:3964-74.

67. Yoshida T, Kaestner KH, Owens GK (2008) Conditional deletion of Kruppel-like factor 4 delays downregulation of smooth muscle cell differentiation markers but accelerates neointimal formation following vascular injury. *Circ Res.* 102:1548-57.
68. Mazzanti CM*, et al.* (2004) Early genetic mechanisms underlying the inhibitory effects of endostatin and fumagillin on human endothelial cells. *Genome Res.* 14:1585-93.
69. Liao X*, et al.* (2011) Kruppel-like factor 4 regulates macrophage polarization. *J Clin Invest.* 121:2736-49.
70. Takizawa H*, et al.* (2010) Lnk regulates integrin alphaIIbbeta3 outside-in signaling in mouse platelets, leading to stabilization of thrombus development in vivo. *J Clin Invest.* 120:179-90.
71. Kwon SM*, et al.* (2009) Pivotal role of lnk adaptor protein in endothelial progenitor cell biology for vascular regeneration. *Circ Res.* 104:969-77.
72. Fujioka S*, et al.* (2004) NF-kappaB and AP-1 connection: mechanism of NF-kappaB-dependent regulation of AP-1 activity. *Mol Cell Biol.* 24:7806-19.
73. Tzima E*, et al.* (2005) A mechanosensory complex that mediates the endothelial cell response to fluid shear stress. *Nature* 437:426-31.
74. Carmeliet P*, et al.* (1999) Targeted deficiency or cytosolic truncation of the VE-cadherin gene in mice impairs VEGF-mediated endothelial survival and angiogenesis. *Cell* 98:147-57.
75. Greenlee MC, Sullivan SA, Bohlson SS (2008) CD93 and related family members: their role in innate immunity. *Curr Drug Targets* 9:130-8.
76. Venkatesh D*, et al.* (2011) RhoA-mediated signaling in Notch-induced senescence-like growth arrest and endothelial barrier dysfunction. *Arterioscler Thromb Vasc Biol.* 31:876-82.
77. Zhu X, Hu C, Zhang Y, Li L, Wang Z (2001) Expression of cyclin-dependent kinase inhibitors, p21cip1 and p27kip1, during wound healing in rats. *Wound Repair Regen.* 9:205-12.
78. Thompson LJ, Jiang J, Madamanchi N, Runge MS, Patterson C (2001) PTP-epsilon, a tyrosine phosphatase expressed in endothelium, negatively regulates endothelial cell proliferation. *Am J Physiol.* 281:H396-403.
79. Grove AD*, et al.* (2002) Both protein activation and gene expression are involved in early vascular tube formation in vitro. *Clin Cancer Res.* 8:3019-26.
80. Zheng PP, van der Weiden M, Kros JM (2007) Hela l-CaD is implicated in the migration of endothelial cells/endothelial progenitor cells in human neoplasms. *Cell Adh Migr.* 1:84-91.
81. Dekker RJ*, et al.* (2006) KLF2 provokes a gene expression pattern that establishes functional quiescent differentiation of the endothelium. *Blood* 107:4354-63.
82. Trojanowska M (2000) Ets factors and regulation of the extracellular matrix. *Oncogene* 19:6464-71.
83. Wasserman SM*, et al.* (2002) Gene expression profile of human endothelial cells exposed to sustained fluid shear stress. *Physiol Genomics* 12:13-23.
84. Perez-Gomez E*, et al.* (2010) The role of the TGF-beta coreceptor endoglin in cancer. *TheScientificWorldJOURNAL* 10:2367-84.
85. van Laake LW*, et al.* (2006) Endoglin has a crucial role in blood cell-mediated vascular repair. *Circulation* 114:2288-97.
86. DeLisser HM*, et al.* (1997) Involvement of endothelial PECAM-1/CD31 in angiogenesis. *Am J Pathol.* 151:671-7.
87. Miragliotta V, Ipina Z, Lefebvre-Lavoie J, Lussier JG, Theoret CL (2008) Equine CTNNB1 and PECAM1 nucleotide structure and expression analyses in an experimental model of normal and pathological wound repair. *BMC Physiol.* 8:1.
88. Isenberg JS, Martin-Manso G, Maxhimer JB, Roberts DD (2009) Regulation of nitric oxide signalling by thrombospondin 1: implications for anti-angiogenic therapies. *Nat Rev Cancer* 9:182-94.
89. Matsuba M, Hutcheon AE, Zieske JD (2011) Localization of thrombospondin-1 and myofibroblasts during corneal wound repair. *Exp Eye Res.* 93:534-40.
90. Huang CJ*, et al.* (2011) A predicted protein, KIAA0247, is a cell cycle modulator in colorectal cancer cells under 5-FU treatment. *J Transl Med.* 9:82.

91. Fukushi J*, et al.* (1998) Novel biological functions of interleukin-4: formation of tube-like structures by vascular endothelial cells in vitro and angiogenesis in vivo. *Biochem Biophys Res Commun.* 250:444-8.
92. Volpert OV*, et al.* (1998) Inhibition of angiogenesis by interleukin 4. *J Exp Med.* 188:1039-46.
